# RGG-motif protein Scd6 affects oxidative stress response by regulating cytosolic caTalase T1 (Ctt1)

**DOI:** 10.1080/15476286.2026.2613892

**Published:** 2026-01-09

**Authors:** Sweta Tiwari, Chitra Togra, Sudharshan Sj, Purusharth I Rajyaguru

**Affiliations:** a Department of Biochemistry, Indian Institute of Science, Bangalore, India; b Department of Physiology, University of Tennessee Health Science Center, Memphis, India

**Keywords:** Post-transcriptional control, catalase, Scd6, reactive oxygen species, oxidative stress

## Abstract

In response to stress, cells undergo gene expression reprogramming to cope with external stimuli. Cells utilize a conserved stress response mechanism called global downregulation of translation, leading to the storage of translationally repressed mRNAs in RNA granules. During oxidative stress induced by H2O2, genes responsible for combating oxidative stress, such as catalases, are strongly induced. However, the post-transcriptional regulatory events affecting these genes during H2O2 stress are not well-explored. Scd6, an RGG-motif-containing protein in yeast, acts as a translational repressor through its interaction with eIF4G1. This study identifies the role of Scd6 in oxidative stress response by regulating cytoplasmic catalase T1 (CTT1). We observe that peroxide stress induces the assembly of Scd6 puncta, which do not colocalize with P-bodies or stress granules. Scd6 overexpression increased sensitivity, while deletion enhanced tolerance to H2O2 treatment. Increased ROS accumulation and decreased Ctt1 protein levels were observed upon Scd6 overexpression due to translation repression of CTT1 mRNA. CTT1 mRNA interacts with Scd6. smFISH analysis and RNA immunoprecipitation studies reveal that localization of Scd6 to puncta upon peroxide stress reduces its interaction with CTT1 mRNA, allowing derepression. The role of Scd6 in peroxide stress response is conserved since the human homolog LSm14A also localizes to puncta upon H2O2 stress, and its overexpression reduces survival in response to peroxide stress. Overall, this study identifies a unique example of translation regulation whereby stress-induced localization of the translation repressor protein to puncta leads to derepression of the target mRNA.

## Introduction

Under stress conditions, reprogramming gene expression is necessary for a cell to respond to external stimuli. As translation is a highly complex and energy-intensive process, its regulation under stress conditions is crucial for the cell. Global downregulation of translation is a conserved stress response mechanism employed by cells to re-orient their gene expression programmes [1,2]. Translationally repressed mRNAs are often stored in RNA-protein complexes known as RNA granules or RNP condensates [3], which are conserved and have dynamic structures. Upon various stresses such as glucose starvation, heat shock and nitrogen starvation, cells form membrane-less cytoplasmic bodies such as stress granules or P bodies [4–8]. Cytoplasmic RNA granules are sites of mRNA storage and degradation [4].

Upon H_2_O_2_-induced oxidative stress, there is a strong induction of genes involved in combating oxidative stress such as catalases [5] and glutathione peroxidase. These enzymes can neutralize H_2_O_2_^6^. While previous studies have established correlations between transcriptional changes and H_2_O_2_ stress [2,7–9], post-transcriptional regulatory events remain poorly explored. Catalase is a haem-containing antioxidant enzyme that is induced in response to oxidative stress, heat shock and starvation [10,11]. In yeast, cytoplasmic catalase is coded by *CTT1* (Cytoplasmic CaTalase T1) gene and peroxisomal catalase is encoded by the *CTA1*(Catalase A) gene [12]. Transcriptional regulation of *CTT1* has been reported by proteins such as Msn2p/Msn4p, Hog1p, Hap1p, Yap1p, and Zap1p, which act on upstream activating elements (UAS) in the *CTT1* promoter [11,13,14].

RNA-binding proteins play a key role in mediating various steps required for the translational regulation. RGG motif proteins are an emerging and second-largest class of RNA binding proteins [15,16]. These proteins are characterized by the presence of Glycine and Arginine repeats, also termed RGG/RG Box [16]. RGG motif proteins play functional roles in various physiological processes such as transcription, pre-mRNA splicing, DNA damage, regulation of apoptosis and mRNA translation [16]. Apart from interacting with RNA, the RGG-motif proteins can also interact with proteins [16]. Often RGG-motifs are fused to canonical RNA and protein interaction domains, which enhance their ability to regulate mRNA fate [17,18].

Scd6, an RGG motif-containing protein in *S. cerevisiae*, represses translation by preventing the formation of the 43S pre-initiation complex by binding to eIF4G via its RGG-motif [19]. However, the integrity of the cap-binding complex is not lost during repression which suggests that the repressed mRNA can re-enter translation in response to specific physiological cues. Arginine methylation of the RGG-motif augments the interaction between Scd6 and eIF4G, which enhances the translation repression [20]. Scd6 binds itself in RGG-motif dependent manner and self-association regulates its repression activity [21]. Although Scd6 has important roles as a translational repressor and an indirect decapping activator [22], knowledge about its impact and detailed analysis of specific mRNA targets remains unknown. Interestingly, RNA-Seq analysis of polysome-associated mRNAs in the *scd6∆* strain suggests an increased translational efficiency of *CTT1 mRNA* [23].

In this study, we explored the role of Scd6 in the oxidative stress response by translational regulation of *CTT1*. Scd6 represses *CTT1* mRNA translation under-unstressed conditions via its interaction. However, the peroxide treatment reduces this interaction as it induces localization of Scd6 to puncta, devoid of *CTT1* mRNA, leading to its derepression. Our results identified a physiological role for Scd6 in the oxidative stress response, which has been extended to the human homolog LSm14A.

## Results

### Scd6 localizes to puncta in response to H_2_O_2_ stress

**Figure 1.**
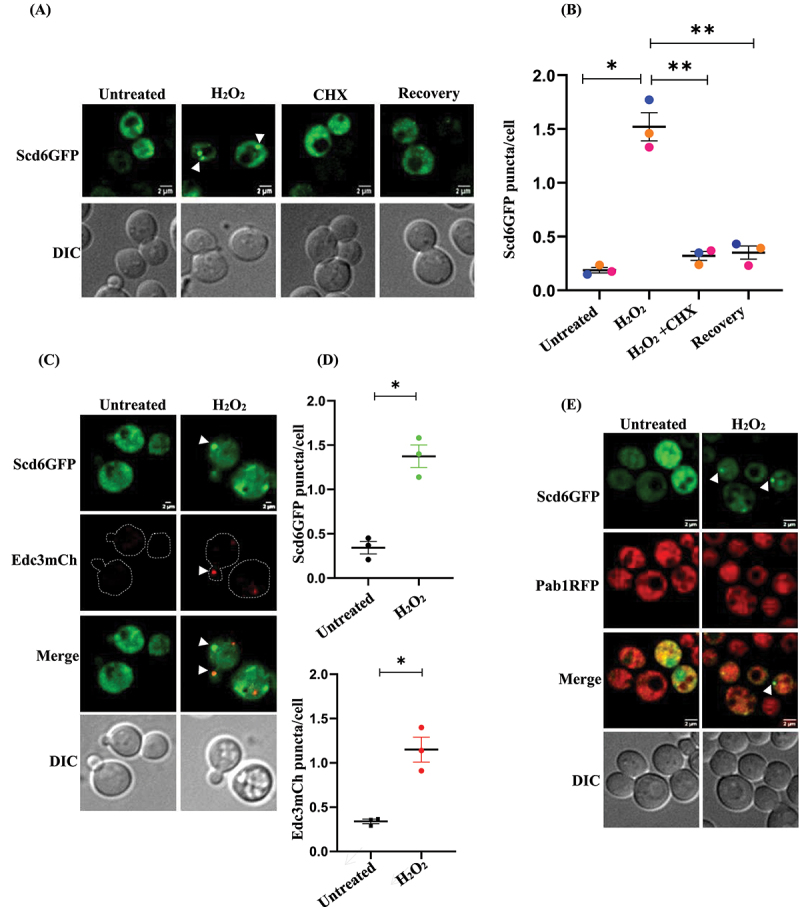
Scd6 localizes to cytoplasmic puncta upon H_2_O_2_ stress- (A) Live cell imaging of WT cells transformed with pRS316 Scd6-GFP plasmid in untreated, 4 mM H_2_O_2,_CHX and recovery conditions (60 minutes) (B) Quantification for the Scd6 puncta formation in (A), (C) Live cell imaging of Scd6-GFP strain transformed with pRS416 Edc3mCh in untreated, 4 mM H_2_O_2_ treated condition (D) Quantification for Scd6-GFP, Edc3mCherry puncta formation, (E) Live cell imaging of Pab1RFP cells transformed with pRS316 Scd6GFP plasmid in untreated, 4 mM H_2_O_2_ treated condition. Data plots represent mean ± SEM from *n* = 3, where ‘*n*’ represents number of independent experiments. Two tailed paired student’s *t*- test was used to calculate the statistical significance ‘*’. Asterisks indicate levels of statistical significance: *p* < 0.05 (*), *p* < 0.01 (**).

Scd6 is known to localize to stress granules and P bodies upon glucose starvation, genotoxic stress and sodium azide stress [19,24,25]. We hypothesized that Scd6 might localize to cytoplasmic puncta in response to other stresses; therefore, we tested the localization of Scd6 in response to 4 mM H_2_O_2_ using live-cell imaging. We observed that Scd6 localized to the puncta upon treatment with 4 mM H_2_O_2_ (Figure 1A,B). To understand whether the Scd6 puncta contained mRNA, we examined the impact of cycloheximide treatment on Scd6 granules. Cycloheximide (CHX) is a well-established inhibitor of translation elongation that stabilizes polysomes by stalling ribosomes on mRNAs, thereby preventing the release of non-translating mRNAs into the cytoplasm which is an essential prerequisite for RNA granule formation [26]. The Scd6 puncta that formed in response to H_2_O_2_ treatment disappeared in response to CHX treatment (Figure 1A,B), suggesting that puncta likely contains mRNA. We observed that the number of Scd6 puncta formed in response to H_2_O_2_ stress decreased upon transferring the cells to H_2_O_2_-free medium (recovery), suggesting that these puncta are potentially dynamic (Figure 1A,B). Figure 1B has also been re-plotted with error bars representing ± SD, and these versions are included in Supplementary Figure S1A. For improved clarity and representation, the middle sections are presented throughout the manuscript. Additionally, the projected images corresponding to Figure 1A, along with their quantification, are provided in Supplementary Figure S1B and S1C.

To determine the identity of the Scd6 puncta formed in response to H_2_O_2_ treatment, we examined the colocalization of Scd6 with Edc3 (core P body marker) and Pab1 (SG marker). Edc3 localized to the puncta upon H_2_O_2_ treatment [26]. We observed the induction of Scd6 and Edc3 puncta upon 4 mM H_2_O_2_ treatment (Figure 1C,D). Figure 1D has also been re-plotted with error bars representing ± SD and is included in Figure S1D. Co-localization experiments between Scd6 and Edc3 (a P-body marker) indicated that Scd6 localized very poorly to P-bodies upon H_2_O_2_ treatment (Figure S1E). To further test whether Scd6 granules formed upon H_2_O_2_ treatment were stress granules, co-localization experiments were performed between Scd6 and the stress granule marker, Pab1. Live-cell imaging was performed on a Pab1RFP strain transformed with pRS316 Scd6GFP plasmid. The H_2_O_2_ treatment did not induce Pab1 puncta formation (Figure 1E), although Scd6 puncta were visible. This is consistent with previous observations where H_2_O_2_ treatment did not induce stress granules in *S. cerevisiae* [26]. To assess whether the kinetics of stress granule formation are delayed under H_2_O_2_ treatment, cells were exposed to H_2_O_2_ for extended durations to monitor the temporal dynamics of granule assembly. Pab1-containing stress granules were not detected, even after prolonged exposure to hydrogen peroxide for up to 90 min (Figure S1F). In contrast, Scd6 displayed more punctate structures with longer treatment durations, as shown in Figure S1F. The change in the localization of Scd6 to dynamic cytoplasmic puncta provided an important clue about the possible role of Scd6 in response to H_2_O_2_ stress. We examined two additional markers to further assess the identity of the Scd6 puncta induced upon H_2_O_2_ stress: Pub1 (a stress granule marker) and Lsm1 (a core P-body marker). Consistent with previous reports that stress granules are not formed during H_2_O_2_ stress, Pub1 also failed to form puncta (Figure S4G). Lsm1 did not show puncta induction under these conditions, whereas Scd6 did (Figure S4H).

### Modulation of Scd6 levels influences cellular tolerance to H_2_O_2_-induced oxidative stress

**Figure 2.**
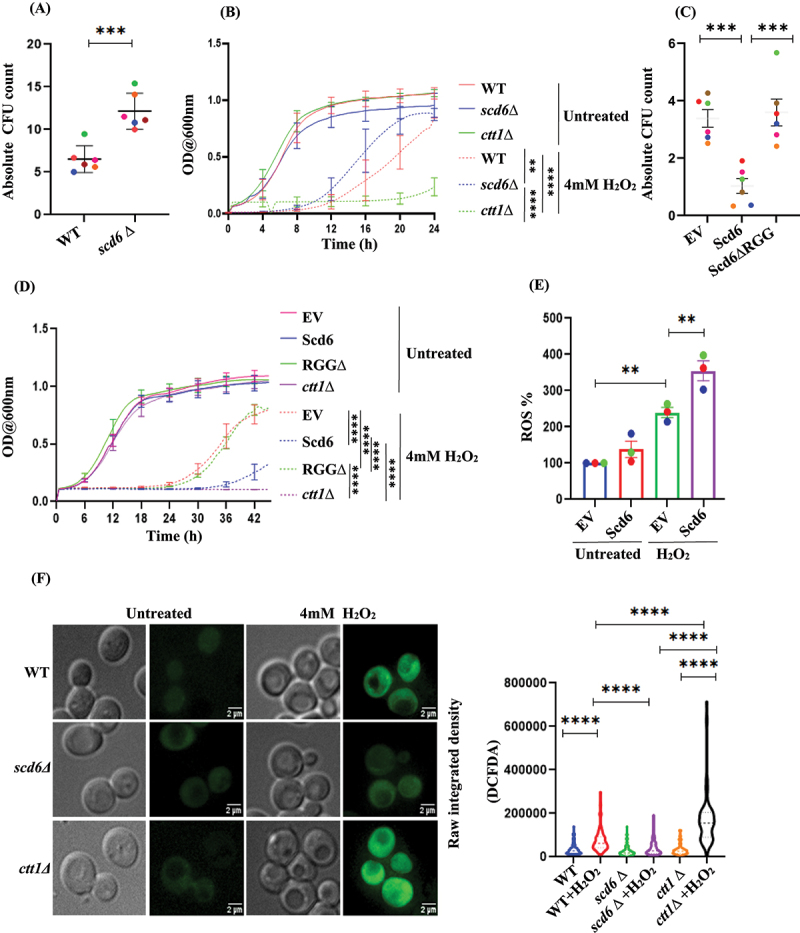
Modulation of Scd6 levels influences cellular tolerance to H_2_O_2_-induced oxidative stress -mid-log phase WT cells and *scd6∆* treated with 4 mM H_2_O_2_ for 30 minutes were plated and scored for their absolute CFU count (treated/untreated) (A) CFU count assay (B) Growth curve showing the survival of WT, *scd6∆* and *ctt1∆*. both the CFU count assay and the growth curve analysis were performed under same conditions (p < 0.0001 for WT vs. WT H_2_O_2,_ *scd6∆* vs. *scd6∆* H_2_O_2_ and *ctt1∆* vs. *ctt1∆* H_2_O_2_). (C) CFU count assay (D) Growth curve showing the survival of WT strain transformed with pYES EV/Scd6GST/Scd6GST*∆*RGG and *ctt1∆* upon 4 mM H_2_O_2_ treatment. Data plots represent mean ± SEM for *n* = 6. Two-tailed paired student’s *t*- test was used to calculate the statistical significance ‘*’ for the CFU count assays. Tukey’s test for variance was used to calculate the statistical significance ‘*’ for growth curve-experiments (n = 4) (p < 0.0001 for EV vs. EV H_2_O_2,_ Scd6GST vs. Scd6GST H_2_O_2,_ Scd6GST*∆*RGG vs. Scd6GST*∆*RGG H_2_O_2_ and *ctt1∆* vs. *ctt1∆* H_2_O_2_) (E) Quantification for relative ROS % upon Scd6 overexpression in untreated and H_2_O_2_ treated condition. Absolute intensities were not plotted due to variability in ROS intensity ranges across experiments (F) Live-cell imaging for DCFDA stained yeast cells for the relative ROS % in WT, *scd6∆* and *ctt1*∆. Violin plot depicting the raw integrated density plotted for the DCFDA signal. Data plots represent mean ± SEM from *n* = 3, where ‘*n*’ represents number of independent experiments. One-way ANOVA was used to calculate the statistical significance ‘*’. Asterisks indicate levels of statistical significance: *p* < 0.05 (*), *p* < 0.01 (**), *p* < 0.001 (***), and *p* < 0.0001 (****).

Since Scd6 was localized to puncta upon H_2_O_2_ treatment (Figure 1), we wondered whether Scd6 plays a role in H_2_O_2_-induced oxidative stress. To test the physiological role of Scd6 in oxidative stress, we performed a colony-forming unit (CFU) count assay and a growth curve analysis of the *scd6∆* strain in the presence of 4 mM H_2_O_2_. From the colony-forming unit count assay, we observed that *scd6∆* cells were more tolerant to 4 mM H_2_O_2_ stress than wild-type cells (Figure 2A). Growth curve analysis of wild type (WT) and *scd6∆* confirmed this observation (Figure 2B). Growth curve analysis was performed across a range of H_2_O_2_ concentrations from 1 to 4 mM (Figure S2 A-C and Figure 2B). Notably, among the tested concentrations, an increased tolerance of *scd6Δ* cells was observed at 4 mM H_2_O_2_ (Figure 2B). In contrast, the overexpression of Scd6 from a 2µ plasmid rendered cells more sensitive to oxidative stress, as demonstrated by the colony-forming unit (CFU) assay (Figure 2C) and growth curve analysis (Figure 2D). This sensitivity appeared to be dependent on the RGG motif of Scd6, as the Scd6ΔRGG mutant did not exhibit the same H_2_O_2_-induced sensitivity as observed with the full-length protein (Figure 2C,D). For the growth curve assays, we used the *ctt1∆* strain as a control, which is known to exhibit hypersensitivity to peroxide treatment [5]. Consistent with the previous observations, *ctt1∆* strain showed sensitivity to H_2_O_2_ treatment (Figure 2B,D). Growth curve analysis was conducted for 1–4 mM H_2_O_2,_ and at all the tested concentrations, overexpression of Scd6 increased the sensitivity of cells to H_2_O_2_ stress in a manner dependent on its RGG motif (Figure S2 D-F and Figure 2D). Based on these results, it appears that the modulation of Scd6 levels influences the cellular response to H_2_O_2_-induced stress.

### Reactive oxygen species levels are modulated by Scd6 in response to H_2_O_2_ treatment

H_2_O_2_ stress leads to increased levels of cellular reactive oxygen species (ROS). The increased sensitivity to H_2_O_2_ stress in cells overexpressing Scd6 from a 2µ plasmid indicated that ROS levels could increase under the same conditions. To test this hypothesis, we performed DCFDA staining to assess ROS levels. Upon H_2_O_2_ treatment, we observed an increased ROS accumulation in cells overexpressing Scd6 compared to those carrying the empty vector control (Figure 2E). Conversely, live-cell imaging following DCFDA staining of *scd6∆* cells indicated significantly reduced accumulation of ROS compared to the WT cells upon H_2_O_2_ exposure (Figure 2F). This observation was consistent with the fluorescence intensity measurements obtained using a plate reader (data not shown). The *ctt1*∆ strain was used as a positive control to confirm ROS accumulation under H_2_O_2_ stress conditions [12,27,28] (Figure 2F). This result suggests a possible role of Scd6 in modulating changes in ROS accumulation in response to H_2_O_2_ treatment.

### Scd6 modulates Ctt1 protein levels

Catalase plays an important role in mitigating H_2_O_2_ stress. A high-throughput study suggested that Cytosolic CaTalse T1 (*CTT1*) mRNA had more ribosome occupancy and translational efficiency in *scd6∆* mutant without significant change in mRNA stability [23]. Therefore, we hypothesize that the observed changes in ROS levels under *scd6∆* and Scd6 overexpression conditions may be associated with alterations in Ctt1 protein levels.

**Figure 3.**
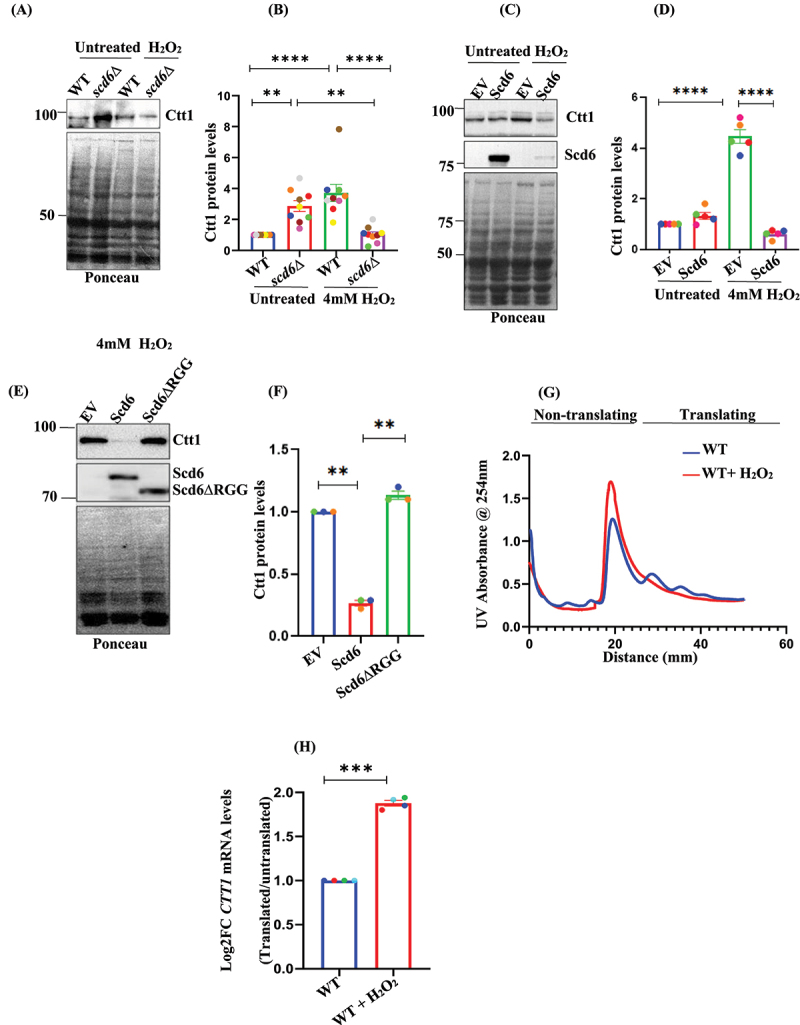
Scd6 affects Ctt1 levels via its RGG-motif- (A) Ctt1-GFP protein levels in WT and *scd6∆* under untreated and 4 mM H_2_O_2_ condition (B) Quantification for (A). Data plots represent mean ± SEM from *n* = 5. Data plots represent mean ± SEM from *n* = 9. (C) Ctt1-GFP protein levels upon Scd6GST overexpression in untreated and 4 mM H_2_O_2_-treated (30 minutes) (D) Quantification for Ctt1 protein levels in (C) Data plots represent mean ± SEM from *n* = 5. Data plots represent mean ± SEM from *n* = 7 (E) Ctt1-GFP protein levels upon Scd6GST/Scd6GST∆RGG overexpression upon 4 mM H_2_O_2_ treatment for 30 minutes (F) Quantification for (E). The GFP, GST signal was detected using α-GFP, α-GST antibody. Ponceau was used as a loading control. Data plots represent mean ± SEM from *n* = 3 (G) Polysome profile analysis of WT cells under untreated and H_2_O_2_-treated conditions. (H) Log_2_ fold-change (log_2_fc) values depicting the distribution of *CTT1* mRNA across polysomal fractions in WT cells. Data are presented as mean ± SEM from *n* = 4 independent experiments. Statistical significance was assessed using a one-way ANOVA. Asterisks indicate levels of statistical significance: *p* < 0.05 (*), *p* < 0.01 (**), *p* < 0.001 (***), and *p* < 0.0001 (****).

Scd6 functions as a translational repressor, and one possible mechanism by which it may influence Ctt1 levels is through repression of its translation. If Scd6 play a role in repressing *CTT1* translation in the absence of stress, then it could be anticipated that Ctt1 levels would increase in the absence of Scd6 due to the derepression of *CTT1* mRNA. Therefore, we checked Ctt1GFP protein levels in *scd6∆*, which were observed to be higher than the WT in mid-log phase cells (Figure 3A,B). After H_2_O_2_ treatment, we observed the induction of Ctt1 in WT but not in *scd6∆*. The absolute values for Figure 3A,B are replotted in Figure S3A. Under these conditions, there was no significant difference in *CTT1* mRNA levels between WT and *scd6∆* (Figure S3B). These findings suggested that Scd6 may contribute to the H_2_O_2_ stress response, possibly by modulating Ctt1 protein levels.

Since *scd6Δ* cells exhibit increased Ctt1 levels, it is plausible that the overexpression of Scd6GST from a high-copy number plasmid, such as 2µ may contribute to the repression of *CTT1* mRNA. qRT-PCR analysis of SCD6 mRNA levels confirmed the overexpression of Scd6 under Scd6GST overexpression condition (Figure S3C). Additionally, to verify the protein expression, a GST-tagged version of Scd6 was introduced into a Scd6-myc background. Western blot analysis comparing this construct to an empty vector control further confirmed the expression of the tagged protein, as shown in Figure S3D. We tested the effect of Scd6 overexpression from a 2µ plasmid on Ctt1 protein levels in 4 mM H_2_O_2_-treated mid-log phase cells. We observed increased protein levels of Ctt1 upon exposure to H_2_O_2_ stress in WT; however, Scd6 overexpression in cells treated with H_2_O_2_ not only inhibited the induction of Ctt1 levels but also led to a significant decrease in Ctt1 protein levels (Figure 3C,D). Interestingly, Scd6 overexpression did not affect Ctt1 levels in the absence of stress (Figure 3C,D). Notably, we also observed a reduction in Scd6GST levels under conditions of H_2_O_2_ stress (Figure 3C). The absolute data points corresponding to Figure 3D have been replotted and are presented in Figure S3E. No significant difference was observed in *CTT1* mRNA levels upon overexpression of Scd6 with respect to EV (Figure S3F).

Scd6 can repress mRNA translation by binding to eIF4G via its RGG motif, thus inhibiting the formation of the 43S pre-initiation complex [19]. We hypothesized that the RGG motif of Scd6 affects Ctt1 protein levels. Therefore, we checked Ctt1 protein levels upon overexpression of Scd6GST∆RGG and observed that the overexpression of this variant did not decrease Ctt1GFP protein levels similar to the full-length Scd6GST (Figure 3E,F). This indicated that the RGG motif of Scd6 is important for mediating *CTT1* repression. The absolute data points corresponding to Figure 3E have been replotted and are presented in Figure S3G. The overexpression of Scd6GST∆RGG did not sensitize cells to H_2_O_2_ stress unlike full-length Scd6GST, highlighting the critical role of the RGG motif in this response (Figure 2C,D).

*CTT1* levels increased upon exposure to peroxide stress. One possible explanation is that *CTT1* regulation may occur, at least in part, at the translation level in response to H_2_O_2_ stress. To explore this, we examined the association between *CTT1* mRNA and the polysomes under H_2_O_2_-treated conditions. Under untreated conditions, WT cells displayed typical polysome profiles with visible 40S, 60S, 80S, and polysome peaks (Figure 3G). In contrast, H_2_O_2_-treated samples showed a marked collapse of polysomes (Figure 3G), consistent with previous reports that acute oxidative stress disrupts global translation, often through inhibition of the initiation or elongation steps [8]. We observed an increase in *CTT1* mRNA association with polysomes in WT cells upon 4 mM H_2_O_2_ treatment (Figure 3H), suggesting translational upregulation under oxidative stress. This observation indicates that elevated *CTT1* localization to polysomes indicates a stress-induced translational activation of *CTT1* mRNA.

### Scd6 interacts with CTT1 mRNA and this interaction is reduced upon peroxide stress

**Figure 4.**
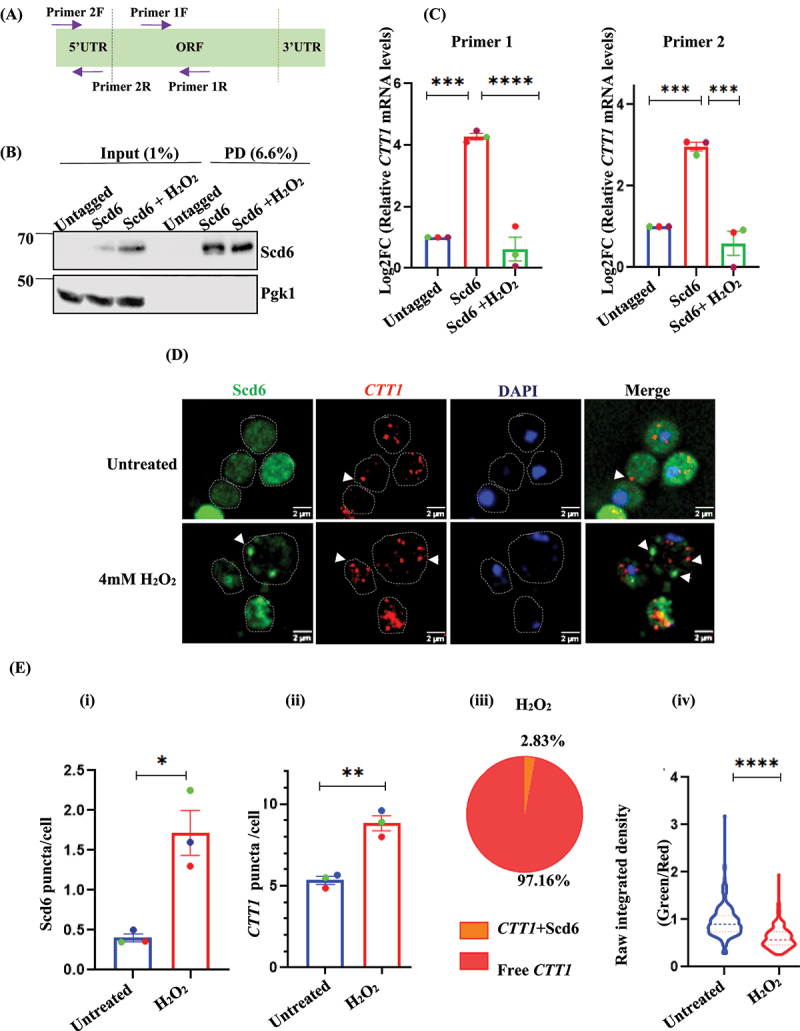
Scd6 interacts with *CTT1* in unstressed conditions and dissociates upon H_2_O_2_ stress-induced granule formation –(A) Schematic for primer positions on CTT1 (B) Blot represents input and PD for endogenous Scd6myc pull-down in untagged and Scd6myc cells. The myc signal was detected using α-myc antibody. PGK1 was used as a control. (C) Log_2_ fold-change (Log2FC) for *CTT1* in the PD fraction showing the enrichment of *CTT1* mRNA in the Scd6myc pull-down using primer 1, primer 2. Statistical significance was assessed using a one-way ANOVA. (D) *CTT1* mRNA was detected using smiFISH (single molecule inexpensive fluorescent in-situ hybridisation) using Cy5 labelled secondary probe. WT cells used were transformed with pRS316 Scd6GFP. (E) quantitation for (D) (i) Scd6GFP puncta/cell (ii) *CTT1* mRNA puncta/cell (iii) pie chart depicting the % colocalization of *CTT1* mRNA with Scd6GFP upon H_2_O_2_ treatment (iv) Raw integrated density quant to depict the *CTT1* mRNA present in the same spatial coordinates as of Scd6GFP in untreated and H_2_O_2-_treated condition. Data plots represent mean ± SEM from *n* = 3, where ‘*n*’ represents number of independent experiments. Paired t-test was used to calculate the statistical significance. Asterisks indicate levels of statistical significance: *p* < 0.05 (*), *p* < 0.01 (**), *p* < 0.001 (***), and *p* < 0.0001 (****).

We observed that in response to 4 mM H_2_O_2_ treatment, there was translational upregulation of *CTT1* mRNA in the polysomes. Since Scd6 affects Ctt1 protein levels without affecting mRNA levels, we hypothesized that Scd6 could interact with *CTT1* mRNA to regulate its translation. To investigate the interaction between Scd6 and *CTT1* mRNA in WT cells under the same conditions, we assessed *CTT1* mRNA binding to endogenous myc-tagged Scd6. RNA immunoprecipitation (RIP) was performed using an anti-myc antibody in a strain expressing Scd6-myc, and the enrichment of *CTT1* was checked using two independent primer pairs targeting different regions of the *CTT1* transcript (Figure 4A). Scd6-myc was enriched in the immunoprecipitated fraction, confirming the efficiency of the pull-down assay (Figure 4B). We detected the enrichment of *CTT1* mRNA in Scd6-myc pull-down cells compared to wild-type cells (Figure 4C). Interestingly, upon treatment with H_2_O_2_, the enrichment of *CTT1* mRNA in Scd6-myc pull-downs was markedly reduced (Figure 4C). This suggests that Scd6–*CTT1* mRNA interaction may be modulated in response to oxidative stress. The absolute values in Figure 4C are shown in Figure S4A. *RAD50*, previously reported to interact with Scd6 [29], served as a positive control, while *ACT1* was used as a negative control. As anticipated, *RAD50* mRNA was enriched (Figure S4B), whereas *ACT1* mRNA was not (Figure S4C). Notably, unlike Scd6 expressed from a 2µ plasmid, endogenous Scd6 protein levels appeared to increase upon peroxide treatment (Figure 3C, Figure S4 D and E).

Scd6 was observed to localize to puncta upon H_2_O_2_ stress (Figure 1A) and under the same conditions its interaction with *CTT1* mRNA appeared to be significantly reduced compared to the unstressed state, where Scd6 is primarily diffused in the cytoplasm (Figure 4C). This observation led us to speculate that the stress-induced localization of Scd6 to puncta might contribute to a reduced interaction with *CTT1* mRNA, potentially facilitating the derepression of *CTT1* mRNA leading to increased translation during H_2_O_2_ stress as observed in the polysome experiment (Figure 3H). We performed (single-molecule inexpensive fluorescence in situ hybridization – smFISH) to test this. In the smiFISH experiments, we observed that Scd6 puncta formed upon peroxide stress largely lacked *CTT1* mRNA signal (Figure 4D and E iii). *ctt1*∆ was used as a control for smFISH experiments to ensure the specificity of *CTT1* smFISH probes (Figure S4F). We observed the absence of any signal in *ctt1∆* cells with *CTT1*-specific probes. In response to the peroxide treatment, we observed an induction of Scd6 puncta formation (Figure 4E i). *CTT1* mRNA expression was also induced under similar conditions (Figure 4E ii). To assess the relative association of Scd6 with *CTT1* mRNA under unstressed conditions, we measured the raw integrated density of the *CTT1* mRNA puncta and the corresponding Scd6 signal at the same spatial coordinates. We observed that the relative association of Scd6 with *CTT1* mRNA decreased after peroxide treatment (Figure 4E iv). Taken together with the previously presented data, these findings suggest a possible mechanism underlying the derepression of *CTT1* mRNA during peroxide stress, which may contribute to the translational induction of Ctt1 and support the peroxide stress response.

Based on the observed translational activation of *CTT1* under H_2_O_2_-induced stress (Figure 3G,H) and the corresponding decrease in its interaction with Scd6 (Figure 4C), we hypothesized that dissociation of *CTT1* mRNA from Scd6 May be a necessary step for its efficient translation under oxidative conditions. Supporting this idea, smiFISH experiments also revealed that Scd6 relocalizes to the cytoplasmic puncta upon stress, and that these granules lack detectable *CTT1* mRNA signals (Figure 4D). We further speculated that if Scd6 targets *CTT1* mRNA, its overexpression might increase the interaction between the two, potentially limiting their stress-induced dissociation and, in turn, influencing *CTT1* translation during oxidative stress. Therefore, we investigated whether this effect might be associated with an interaction between Scd6 (expressed from a 2µ plasmid) and *CTT1* mRNA by RNA immunoprecipitation (RIP) experiments using two independent primer pairs targeting different regions of the *CTT1* transcript (Figure S5A). The enrichment of Scd6 in the pull-down samples (Figure S5B). Under unstressed conditions, we detected enrichment of *CTT1* mRNA in Scd6 pull-downs, suggesting a potential interaction between Scd6 and *CTT1* mRNA (Figure S5C). However, Scd6 protein levels appeared to decrease after H_2_O_2_ treatment (Figure 3C and S5B). Despite this reduction, we observed a marked enrichment of *CTT1* mRNA in Scd6 immunoprecipitates under stress conditions when Scd6 was overexpressed (Figure S5C), suggesting that peroxide stress may enhance the association between Scd6 and *CTT1* mRNA. This enhanced interaction is also correlated with the decreased polysomal association of *CTT1* mRNA upon Scd6 overexpression (Figure S5D). However, further investigation is required to determine whether this interaction is directly and functionally significant in the regulation of *CTT1* expression. To assess the specificity of this interaction, we tested the association of Scd6 with two other mRNAs. *CTA1* and *RAD50*, previously reported to interact with Scd6 [29], served as a positive control, while *ACT1* was used as a negative control. As expected, *CTA1* and *RAD50* mRNA was enriched (Figure S5 E and F), whereas *ACT1* mRNA was not (Figure S5G). To investigate whether the interaction between *CTT1* mRNA and Scd6 depends on the RGG motif – previously implicated in RNA binding and protein–protein interactions, we performed RNA immunoprecipitation (RIP) using strains expressing either full-length Scd6 or the RGG deletion mutant (Scd6ΔRGG). The enrichment of Scd6 in the pull-down fractions confirmed the efficiency of the RIP (Figure S5H). *CTT1* mRNA was enriched in immunoprecipitated from cells expressing full-length Scd6; however, this enrichment was absent in the Scd6ΔRGG samples (Figure S5 I), suggesting that the interaction between Scd6 and *CTT1* mRNA is dependent on the RGG motif.

Since Scd6 has been reported to undergo arginine methylation (AM) [20], we wondered whether the AM status of Scd6 was altered upon peroxide stress, inducing a change in the Scd6-*CTT1* interaction in wild-type cells. We observed a marginal but significant decrease in the mono-methylation of Scd6 after H_2_O_2_ treatment (Figure S6).

### LSm14A (Scd6 human ortholog) contributes to peroxide stress response

**Figure 5.**
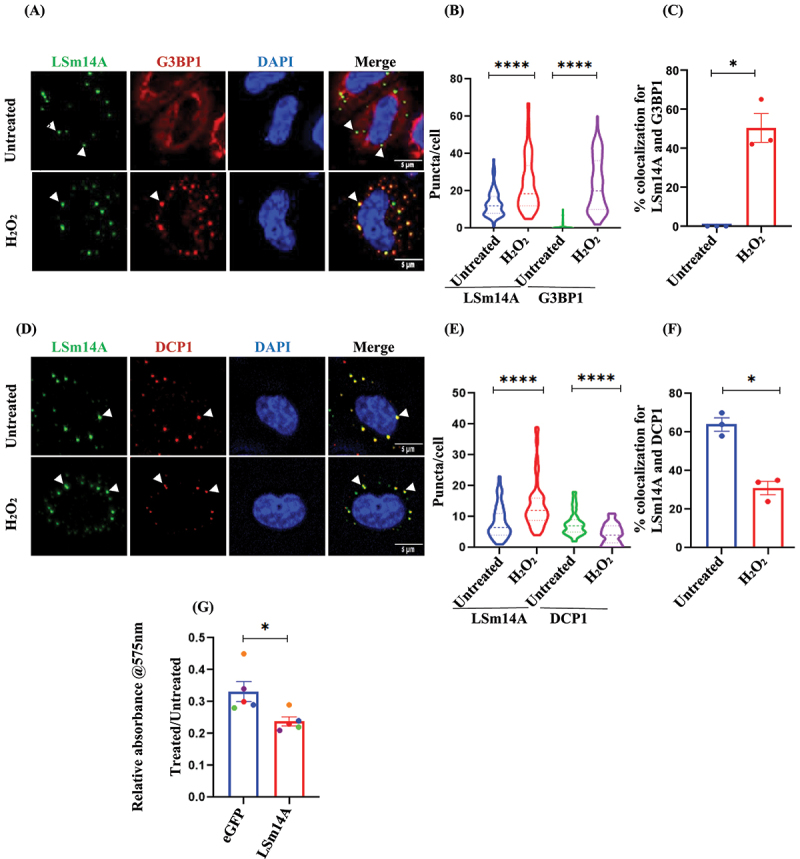
LSm14A localizes to puncta upon oxidative stress (A) Microscopic images of immunostaining of G3BP1 in LSm14AGFP transfected HeLa cells treated with water (vehicle control) and 1 mM H_2_O_2_ for 2 h. Endogenous G3BP1 was detected using α-G3BP1 antibody. (B) Quantification for LSm14AGFP and G3BP1 puncta per cell as in (A). (C) Percentage colocalization of LSm14AGFP puncta with G3BP1 upon H_2_O_2_ treatment. (D) Microscopic images of immunostaining of DCP1 in LSm14AGFP transfected HeLa treated with water and 1 mM H_2_O_2_ for 2 h. Endogenous DCP1 was detected using α-DCP1 antibody. (E) Quantification for LSm14AGFP and DCP1 puncta per cell as in (D). (F) Percentage colocalization of LSm14AGFP puncta with DCP1 upon H_2_O_2_ treatment. Data plots represent mean ± SEM from *n* = 3, where ‘*n*’ represents number of independent experiments. Two tailed paired *t*- test was used to calculate the statistical significance ‘*’. (G) Cell viability assays in eGFP or LSm14AGFP transfected HeLa cells treated with 2 mM H_2_O_2_ for 2 h. Data plots represent mean ± SEM from *n* = 5, where ‘*n*’ represents number of independent experiments. Two tailed paired student’s *t*- test was used to calculate the statistical significance ‘*’. Asterisks indicate levels of statistical significance: *p* < 0.05 (*), *p* < 0.01 (**), *p* < 0.001 (***), and *p* < 0.0001 (****).

Scd6 orthologs have been reported in various model organisms including LSm14A in humans [30]. Therefore, we tested if the role of Scd6 in peroxide stress response is conserved in human cells. Therefore, we performed microscopy to assess LSm14A localization in HeLa cells in response to H_2_O_2_ treatment. Under unstressed conditions, LSm14A was localized to the cytoplasmic puncta. Upon treatment with H_2_O_2_, the number of LSm14A puncta increased (Figure 5A,D), suggesting enhanced recruitment of LSm14A to cytoplasmic puncta in response to oxidative stress. Upon treatment with peroxide, we observed an increase in the formation of G3BP1 puncta (Figure 5B), suggesting enhanced stress granule assembly under oxidative stress conditions. In contrast, the number of DCP1 puncta appeared to decrease under the same conditions (Figure 5D,E), indicating a potential reduction in P-body formation or altered dynamics in response to stress. Colocalization studies with G3BP1 (SG marker) and DCP1 (PB marker) revealed that the colocalization of LSm14A decreased with DCP1 and increases with G3BP1 upon H_2_O_2_ (Figure 5C,F). Overexpression of Scd6 rendered yeast cells more sensitive to H_2_O_2_ treatment (Figure 2C,D). To determine whether LSm14A, the human homolog of Scd6, exhibits similar behaviour, we conducted an MTT assay. The results indicated that overexpression of LSm14A increased the sensitivity of HeLa cells to H_2_O_2_ treatment compared to that of the empty vector control (Figure 5G). Overall, the localization of Scd6 in cytoplasmic granules appears to be conserved; however, the specific context and functional implications of this localization may vary.

## Discussion

In this study, we report on the role of the RNA-binding protein Scd6 in H_2_O_2_ stress response mediated by the *CTT1* mRNA regulation. The key observations supporting this finding are as follows: (1) Scd6 localizes to dynamic puncta upon H_2_O_2_ treatment (Figure 1), (2) *scd6Δ* cells are more tolerant to H_2_O_2_ treatment (Figure 2A,B), whereas Scd6 overexpression increased sensitivity to peroxide stress (Figure 2C,D), (3) There is increased ROS accumulation upon peroxide stress in cells overexpressing SCD6 (Figure 2E), (4) Deletion of SCD6 increases Ctt1 protein levels (Figure 3A,B) in unstressed cells and SCD6 overexpression reduces Ctt1 protein levels in RGG-motif dependent manner in response to H_2_O_2_ treatment (Figure 3C–F) (5) *CTT1* undergoes translational activation in response to H_2_O_2_ treatment (Figure 3G,H) (6) Scd6 interacts with *CTT1* mRNA and this interaction diminishes upon peroxide stress (Figure 4C) (7) Colocalization of *CTT1* mRNA reduces with Scd6 granules upon stress (Figure 4D,E) (8) LSm14A (human homolog of Scd6) localizes to a punctum upon H_2_O_2_ treatment (Figure 5A–F) and (9) LSm14A overexpression increases sensitivity to peroxide stress (Figure 5G).

Scd6 was localized to distinct puncta upon H_2_O_2_ treatment, which decreases after the removal of stress, highlighting their dynamic nature (Figure 1A). The Scd6 puncta observed following H_2_O_2_ treatment were no longer visible after cycloheximide (CHX) treatment (Figure 1A,B), indicating that these puncta may contain RNA. Colocalization experiments suggested that these Scd6 puncta did not colocalise with Edc3 (P-body marker) (Figure 1C,D, and S1E). We did not observe stress granule formation under similar conditions, consistent with previous observations [26] (Figure 1E and S1F), indicating that the Scd6 puncta assembled in response to peroxide stress are perhaps neither PBs nor SGs. Since Scd6 does not colocalize with Edc3 (a P-body marker) and Pab1 puncta (SG marker) are not formed upon H_2_O_2_ treatment, it is possible that Scd6 forms distinct puncta in response to H_2_O_2_-induced stress. Another possibility is that these Scd6 puncta represent a distinct subset of P-bodies and stress granules lacking Edc3 and Pab1, differing in composition from the canonical PBs and SGs. To further evaluate the identity of the Scd6 puncta, we also examined additional markers representing both granule types. Pub1, another stress granule marker, did not form puncta under H_2_O_2_ stress, consistent with the absence of stress granules in this condition. Similarly, the core P-body marker Lsm1 also failed to show puncta induction, whereas Scd6 robustly formed puncta.

Overexpression and deletion of *SCD6*Δ appeared to influence the survival of yeast cells under H_2_O_2_-induced oxidative stress. Although *scd6Δ* cells exhibited a relatively greater tolerance, Scd6 overexpression was associated with increased sensitivity to H_2_O_2_ treatment (Figure 2A–D). These findings suggest a potential role of Scd6 in modulating cellular response to oxidative stress. Growth curve analyses for Scd6 deletion and overexpression were performed across a range of peroxide concentrations (1–4 mM) to assess cellular responses to varying levels of oxidative stress (Figure S2, Figure 2). The sensitivity and tolerance correlate with the status of ROS. Cells overexpressing Scd6 overexpression, which showed sensitivity to H_2_O_2_ treatment, were found to have significantly higher ROS levels (Figure 2E). In contrast, cells with Scd6 deletion displayed reduced ROS levels compared to the WT cells following H_2_O_2_ treatment, suggesting enhanced oxidative stress tolerance in the absence of Scd6 (Figure 2F). This result established the physiological role of Scd6 in response to H_2_O_2_-mediated oxidative stress.

Ctt1 protein levels were elevated in untreated *scd6Δ* cells. However, upon H_2_O_2_ treatment, a reduction in Ctt1 levels was observed in these cells, which may seem counterintuitive (Figure 3A,B). The increased basal levels of Ctt1 in *scd6Δ* cells likely resulted from de-repression owing to the absence of Scd6. One possible explanation is that elevated basal levels of Ctt1 protein may fulfil the need for Ctt1, thereby diminishing the requirement for further induction under oxidative stress. In this context, feedback regulatory mechanisms might restrict additional Ctt1 expression in response to H_2_O_2_, allowing the cell to maintain homoeostasis and preventing excessive or unnecessary protein synthesis. This remains to be tested experimentally in the future. Scd6 is a translational repressor protein. Since Ctt1 is an important enzyme implicated in the peroxide stress response, we tested the impact of Scd6 on Ctt1 protein levels. Ctt1 levels were induced in response to the peroxide stress (Figure 3C,D); however, Scd6 overexpression from a 2µ plasmid inhibits this induction (Figure 3D). We observed that Scd6 overexpression reduced Ctt1 protein levels in an RGG-dependent manner (Figure 3E,F).

Evident changes in Ctt1 protein levels, without corresponding alterations in *CTT1* mRNA levels (Figure S3 A-B and E-F) upon Scd6 deletion or overexpression, suggested the involvement of post-transcriptional regulatory mechanisms. To explore the possibility that *CTT1* mRNA undergoes translational activation in response to oxidative stress, we assessed its translational status using polysome profiling combined with qRT-PCR to determine its association with translationally active and inactive ribosomal fractions. Oxidative stress was induced using 4 mM H_2_O_2_, which is known to trigger acute cellular stress. It is well established that such conditions result in global translational repression, typically characterized by the collapse of polysomes due to the inhibition of translation initiation or stalling of elongation. Consistent with this, our polysome profiles of H_2_O_2_-treated cultures displayed a marked reduction in polysome content, indicative of a widespread translational shutdown (Figure 3G). Despite this global repression, certain stress-responsive mRNAs, particularly those encoding detoxification enzymes and molecular chaperones, can bypass translational inhibition and remain actively translated through alternative mechanisms, including cap-independent initiation, upstream open reading frames (uORFs), and regulation by RNA-binding proteins. Our results indicate that *CTT1* mRNA remains associated with translationally active polysome fractions, even under oxidative stress, supporting the notion of its selective translation under these conditions (Figure 3H).

Endogenous Scd6 levels were found to be upregulated upon treatment with 4 mM H_2_O_2_, suggesting a potential role for Scd6 in the cellular response to oxidative stress induced by this condition (Figure 4B, S4D and E). Under similar oxidative stress conditions that promote translational activation of *CTT1* mRNA, we observed a dissociation of *CTT1* mRNA from Scd6, which was otherwise associated under unstressed conditions (Figure 4C). This stress-induced dissociation suggested that release from Scd6 may be a prerequisite for the translation of*CTT1* mRNA during oxidative stress. This was further confirmed by smFISH analysis, wherein Scd6 puncta induced by peroxide stress largely excluded *CTT1* mRNA (Figure 4D,E). In this experiment, Scd6GFP was expressed from a CEN (pRS316) plasmid whose expression levels were closer to the wild type than the relatively high levels of Scd6 when expressed from 2µ plasmid. Assembly of RNA granules is often associated with translation repression and mRNA storage [31]. *CTT1* mRNA was largely excluded from the Scd6 puncta (Figure 4D,E). One possibility is that Scd6 relocalizes to the cytoplasmic puncta under these conditions, which might contribute to the regulation of other translationally repressed transcripts. An obvious future direction would be to identify mRNAs that colocalize in these puncta. This could provide further insights into the role of Scd6 in peroxide-mediated oxidative stress response beyond derepressing *CTT1* mRNA.

We further hypothesized that if Scd6 targets *CTT1* mRNA, its overexpression could enhance this interaction and potentially hinder the stress-induced dissociation observed under oxidative conditions. Such sustained association may interfere with the selective translational activation of *CTT1* mRNA during oxidative stress, thereby impacting Ctt1 protein expression. Based on the RIP experiments using Scd6 overexpression, we report that *CTT1* mRNA is a binding target of Scd6 (Figure S5C). RNA immunoprecipitation revealed enrichment of *CTT1* mRNA in Scd6 (expressed from a 2µ plasmid) pull-downs under both stressed and unstressed conditions (Figure S5C). Notably, this association was enhanced (Figure S5C) under stress despite the reduced Scd6 protein levels (Figures 3C and 5B). While overexpression artefacts may partly explain the enhanced interaction between Scd6GST and *CTT1* mRNA, additional factors likely contributed to the observed differences between endogenous and overexpressed Scd6. These include potential disruptions in stress-induced post-translational modifications that normally facilitate mRNA release and alter protein stability under oxidative stress – where endogenous Scd6 is stabilized (Figure S4 D and E), whereas Scd6GST appears destabilized (Figure S5B). We speculate that the bulky GST tag on Scd6GST may interfere with certain post-translational modifications that occur in endogenous Scd6, which could potentially contribute to differences in protein stability between the tagged and native forms. Differences in the post-translational modifications (if any) of endogenous Scd6 and overexpression of Scd6GST remain to be experimentally determined. Together, these factors suggest that Scd6GST may not fully mimic the behaviour of endogenous Scd6 under stress conditions. We plan to explore these possibilities in future studies to better understand the mechanisms underlying the differential behaviour of endogenous and overexpressed Scd6 under oxidative stress.

An interesting aspect of the results presented in this work is the switch that allows changes in the Scd6 protein*-CTT1* mRNA interaction. The interaction under unstressed conditions is significantly diminished in response to peroxide stress. This is important as it likely allows derepression of *CTT1* mRNA. Furthermore, the diminished interaction between Scd6 protein and *CTT1* mRNA correlated with reduced Scd6 mono-methylation (Figure S6). The reduction in methylation was not very strong but significant. A limitation of this experiment is that it was carried out in the SCD6 overexpression (2µ plasmid) background. It is important to ascertain the methylation status of endogenous Scd6 in response to H_2_O_2_ stress. We attempted this experiment, however, detection of the methylation status of endogenous Scd6 has been technically challenging due to the low copy number of Scd6 (3200 molecules/cell) [32]. Scd6 is a substrate of conserved and predominant methyltransferase Hmt1, which catalyse both monomethylation and asymmetric dimethylation. In the absence of reliable dimethylation-specific antibodies for yeast proteins, we currently do not know the dimethylation status of Scd6 under unstressed conditions or in response to peroxide stress. This information could provide useful insights. Hmt1 could affect the oxidative stress response by altering Scd6 methylation; however, we have refrained from assessing the phenotype of HMT1 deletion and/or overexpression strains as the phenotype(s) in this strain could be complicated due to methylation changes of several known nuclear and cytoplasmic substrate proteins. Creating mutants of arginine residues that are specifically altered in methylation upon peroxide stress will be our future endeavour.

Scd6 is a conserved protein, and homologs of Scd6 in humans, worms, flies, frogs, and plants have been implicated in translational repression and/or mRNA decay (Table 1). The human homolog od Scd6, LSm14A displayed enhanced localization to puncta upon H_2_O_2_ treatment (Figure 5A,D), and its colocalization analysis using G3BP1 (a stress granule marker) and DCP1 (a P-body marker) revealed a decrease in the overlap of LSm14A with DCP1 and a corresponding increase in colocalization with G3BP1 following H_2_O_2_ treatment (Figure 5C,F). These observations suggest that LSm14A may relocalize from P bodies to stress granules in response to oxidative stress. Based on our observations, the localization of Scd6 to cytoplasmic granules appears to be conserved across species; however, the specificity and context of this localization differ. In yeast, Scd6 forms distinct puncta that do not colocalize with canonical stress granules or P-bodies (Figure 1), whereas in HeLa cells, its human homolog LSm14A, predominantly localizes to stress granules following H_2_O_2_ treatment (Figure 5C). To better understand the nature of these H_2_O_2_-induced Scd6 granules, we aimed to further characterize their RNA content and molecular composition in future studies. The MTT assay results demonstrated that overexpression of LSm14A enhanced the sensitivity of HeLa cells to H_2_O_2_ treatment, as compared to cells expressing the empty vector control (Figure 5G). Despite differences in granule identity, both Scd6 and LSm14A may contribute to increased sensitivity to oxidative stress, indicating a potentially conserved physiological role. To explore whether this sensitivity is associated with translational control mechanisms, we plan to identify and analyse the mRNA targets of LSm14A in future experiments.

**Table 1.** Listing the orthologues of Scd6 family of proteins and their mRNA targets.

Name of the orthologue	Organism	Target mRNA	Pathway involved	Mode of regulation	Reference
Scd6	*S. cerevesiae*	*GTO3, LCL1, HVG1, PRM7, RTN2, FMP45, BSC5, PGM2, CTT1, CRS5, GCY1, RTC3, TSL1, TOS8*	Translation, mRNA decay	Translation repression, Decapping activator	Ziedan et al. [23], Nissan et al. [22],
Sum2	*S. pombe*	N/A	mRNA decay	Decapping activity	Fromm et al. [33],
SCD6	*T.brucei*	N/A	mRNA decay/storage	Localizes to RNA granules	Kruger et al. [34]
CAR-1	*C.elegans*	2RSSE.1 aagr-2 aak-2 aat-5 abcf-2 abts-1 abts-3 acc-4 ace-3	Translation, mRNA storage, Intacellular trafficking	Translation repression and mRNA storage	Squirell et al. [35], Tang et al. [36],
TraI	*D.melanogaster*	*sar1*	Translation, Intracellular protein trafficking, endocytosis	Translation repression and mRNA storage	Wilhem et al. [37],
DCP5	*A.thaliana*	*EXPL-1, SEN1, DCP2*	Translation, mRNA decay	Translation repression, mRNA depcapping	Xu and Chua [38],
xRAP55	*X. laevis*	N/A	Translation	Translation repression	Tanaka et al, [39]
Lsm14	*M.musculus*	N/A	Mieosis	Mieotic progression	Zhang et al, [40]
LSm14A/hRAP55	*Human*	*FAM111B*, *LIG4*, *SASS6*	mRNA storage, Mitotic sindle formation	mRNA storage, Stablizes the mitotic spindle	Mili, Georgesse, and Kenani, [41]

**Figure 6.**
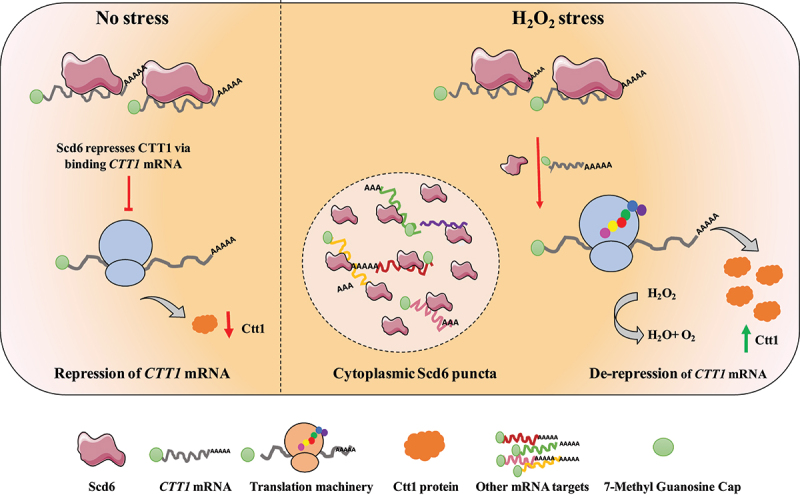
Proposed model for the de-repression of *CTT1* upon peroxide stress.

Overall, this study identifies a unique mechanism underlying the translation regulation of *CTT1* mRNA by Scd6 (Figure 6). Under normal conditions, *CTT1* mRNA binds to Scd6 leading to its repression. In response to peroxide stress, Scd6 assembles indistinct puncta from which *CTT1* mRNA is largely excluded due to reduced *CTT1*-Scd6 interactions. This likely allows derepression of *CTT1* mRNA and increases Ctt1 protein levels, allowing the cells to mount an oxidative stress response.

## Materials and methods

### Yeast strains and plasmids

The yeast strains used in this study were BY4741 and its derivatives (Table 2). These strains were grown either in yeast extract/peptone media or synthetic dropout minimal medium at 30°C 220 rpm. The plasmids used in the study have been listed in Table 3.

**Table 2.** The strains used in the study.

Strain number	Name	Description
yPIR1	Wild type	MATa his3Δ1 leu2 met15 ura3 (BY4741)
yPIR24	*scd6∆*	MATa his3Δ1 leu2 ura3 his3 met15 *scd6*∆::KanMX
yPIR71	Ctt1-GFP	MATa his3Δ1 leu2 met15 ura3 Ctt1-GFP
yPIR13	Scd6-GFP	MATa leu2 ura3 his3 met15 SCD61-GFP (HIS)
yPIR72	Ctt1-GFP *scd6∆*	MATa his3Δ1 leu2 met15 ura3 Ctt1-GFP *scd6*∆::KanMX
yPIR104	Pab1RFP	A gift from Prof. Beidong Liu’s Lab
yPIR153	*ctt1∆*	MATa his3Δ1 leu2 ura3 his3 met15 *ctt1*∆::KanMX

**Table 3.** List of the plasmids used in the study.

Plasmid number	Name	Description
pPIR95	pYES EV	2µ empty vector, Ura and Amp^R^ selection marker
pPIR97	pYES Scd6GST	Expressing Scd6GST under its own promoter, Ura selection marker
pPIR180	pYES Scd6GST∆RGG	Expressing Scd6GST∆RGG under its own promoter, Ura selection marker
pPIR51	pRS316 Scd6GFP	CEN plasmid expressing Scd6GFP, Ura and Amp^R^ selection marker
pPIR130	pGP EV	pGP564,2µ empty vector, Leu- selection marker
pPIR168	pGP Scd6GFP	pGP-Scd6GFP,Expressing Scd6GFP under its own promoter, Leu and Kan^R^ selection marker
pPIR52	pRS416 Edc3mCh	Expressing Edc3mCh, Ura selection marker
pPIR308	peGFP	peGFP-C1, empty vector for mammalian expression
pPIR98	pLSm14aGFP	Expressing LSm14AGFP under pCMV promoter, Amp^R^ selection marker

### Yeast transformation

WT cells were diluted from an overnight grown primary culture to 0.1 OD_600_ and grown to 0.6 OD_600._ The cells were pelleted at 5000 rpm. The pellets were washed twice with water followed by washing with 100 mM LiAc (2 times). After washing, the cells were resuspended in 300 μl of 100 mM LiAc and further aliquoted into three different microcentrifuge tubes. To the cell suspension, 240 μl of 50% PEG (v/v), 36 μl of 1 M LiAc, and 25 μl of salmon sperm DNA (100 mg/ml) were added individually and vortexed vigorously for 15 s. Template DNA was not added to the control. The mixture was then incubated at 30°C for 30 min, followed by 15 min of heat shock at 42°C. The cells were then pelleted. The pellet was resuspended in 100 μl of H_2_O and plates on SD Ura-/SD Leu- plates and incubated at 30°C for 2 days [42].

### H_2_O_2_ treatment

Yeast cells were diluted to an OD_600_ of 0.1 from an overnight grown primary culture and allowed to grow till 0.4–0.8 OD_600_. These cells were then treated with 4 mM H_2_O_2_ for 30 min at 30°C and 220 rpm in dark [5,34]. The cells were then pelleted at 4200 rpm for 1 min at room temperature and stored at −80°C until further use.

### Colony-forming unit (CFU) assay

The yeast cells were diluted to an OD_600_ of 0.1 from an overnight-grown primary culture and allowed to reach 0.8 OD_600_. After it reached 0.8 OD_600_, the cultures were split into two parts: to one part, 4 mM H_2_O_2_ was added, and an equal amount of water was added to the other half of the culture. Both were incubated at 30°C for 30 min at 220 rpm. The cells were then serially diluted, followed by plating different dilutions on yeast extract/peptone media and synthetic dropout minimal media agar plates. The plates were incubated at 30°C for 2 days. The colony forming unit (CFU) count was used to determine survival [34]. The percentage survival was normalized to that of the untreated cells. Statistical analysis was performed using GraphPad Prism Version 8.0.

### Growth curve assay

Yeast cells were diluted from an overnight-grown primary culture at an initial OD_6__00_ of 0.1 and grown until they reached mid-log phase (0.8 OD_6__00_). At this point, the cultures were divided into two equal parts: one was treated with 4 mM H_2_O_2_, while the other received an equal volume of MQ water as a control. Both cultures were incubated at 30°C for 30 min with shaking at 220 rpm. Following treatment, cells were adjusted to an OD_6__00_ of 0.1, serially diluted, and inoculated into 96-well plates at 30°C. OD_6__00_ readings were recorded every 30 min for 24-48 h using a Tecan Infinite Pro 500 plate reader to monitor growth kinetics. A two-tailed paired Student’s *t*- test was used to calculate the statistical significance ‘*’ for the CFU count assays. Tukey’s test for variance was used to calculate the statistical significance ‘*’ for growth curve-experiments

### Detection of Intracellular reactive oxygen species (ROS) using H_2_DCFDA staining

Intracellular ROS levels were monitored using 2,7-dichlorofluorescein diacetate (H_2_DCFDA) staining. WT cells with *scd6∆*/Scd6 of 0.8 OD_600_ were treated with 4 mM H_2_O_2_ for 30 min at 30°C followed by washing with 1× Phosphate Buffer Saline (PBS). These cells were incubated with 100 µM H_2_DCFDA [43] for 30 min followed by washing with 1x PBS. After resuspending the cells in 1× PBS, the cells were imaged using Deltavision RT (Real Time) microscope, and fluorescence was measured at an excitation wavelength of 485 nm and emission wavelength of 524 nm using Tecan Infinite Pro 500.

For the microscopic quantification of ROS, 30 cells were used for each condition. The FITC channel was adjusted at 0.2 s of exposure and 32% transmittance for the DCFDA signal. The Raw integrated intensity was measured and plotted for each cell for three independent experiments as shown in Figure 2F.

### Cycloheximide treatment

Yeast cells were diluted to an OD_600_ of 0.1 in 10 ml SD Ura with 2% glucose from an overnight grown primary culture and allowed to grow till 0.4–0.5 OD_600_. These cultures were divided into two parts, 5 ml culture was treated with 4 mM H_2_O_2_ (2.04 µl of 9.8 M stock), and the same volume of H_2_O was added to another 5 ml culture (untreated) followed by 30 min incubation at 30°C at 220rpm in the dark. The H_2_O_2-_treated culture was washed off using SD Ura-. To the 5 ml of H_2_O_2_ treated culture, either 5 µl of 100 µg/ml cyloheximide [44,45] was added. The cells were incubated for 10 min at 30°C and 220 rpm under dark. These cells were then pelleted and used for live cell imaging.

### Microscopy

For yeast, the cells were pelleted at 10,000 rpm for 15 s, spotted on glass cover slip and observed using live cell imaging. Yeast images were acquired using a Deltavision RT microscope system running softWoRx 3.5.1 software (Applied Precision, LLC), using an Olympus 100×, oil immersion 1.4 NA objective. The Green Fluorescent Protein (GFP) channel was adjusted at 0.5 s of exposure and 50% transmittance. The Red fluorescent channel (mCherry) was adjusted at 0.6 s of exposure and 50% transmittance. For the quantification of puncta per cell, we analysed 100 cells/per experiment. Puncta were counted individually for each cell and then normalized to the total number of cells analysed within the imaging field, yielding the *puncta per cell* value. Statistical analyses were performed using GraphPad Prism Version 8.0. Statistical significance was calculated using a two-tailed unpaired/paired t-test. Microscopy experiments shown in Figure S1G and S1H were performed using a Nikon T2 confocal microscope with an exposure time of 300 ms and 50% laser transmittance.

### Polysome profiling

Wild-type BY4741 cells harbouring either an empty vector (EV) or Scd6GST expressed from the pYES plasmid were cultured in 200 mL SD – Ura medium containing 2% glucose until they reached an OD_6__00_ of approximately 0.8. The cells were then treated with 4 mM H_2_O_2_ for 30 min at 30°C in a shaking incubator in the dark conditions. After H_2_O_2_ treatment, the cells were treated with cycloheximide (100 µg/mL) for 15 min at 30°C in a shaking incubator to arrest the translation. Following treatment, cells were rapidly chilled on ice and harvested by centrifugation at 4°C. The pellets were washed with diethyl pyrocarbonate (DEPC) treated, autoclaved Milli-Q water and resuspended in 400 µL of lysis buffer (10 mM Tris pH 7.4, 100 mM NaCl, 30 mM MgCl_2_, 100 µg/mL cycloheximide, 10 U RiboLock RNase inhibitor, 1× Complete Mini EDTA-free protease inhibitor tablet [Roche; 04693132001], and 1 mM PMSF [SRL; 84,375]). Cells were lysed by bead beating with glass beads using a FastPrep homogenizer at 4°C for 15 min (22-s cycles at 4.5 m/s). The obtained lysates were centrifuged at 5500 rpm for 5 min, and the supernatant was collected. Lysate equivalent to approximately 1000 OD_2__54_ units was layered onto a 10–50% sucrose gradient prepared in sucrose gradient buffer (140 mM KCl, 5 mM MgCl_2_, 5 mM Tris-HCl pH 7.5, and 100 µg/mL cycloheximide). The gradients were centrifuged at 39,000 rpm for 2 h at 4°C in a Beckman SW41Ti rotor. Gradient fractions were collected using a BioComp Gradient Profiler. Fractions corresponding to free mRNPs and 80S monosomes were pooled and designated as the untranslated fraction, whereas fractions containing polysomes were pooled and classified as the translated fraction.

### Pull-downs and western blotting

For glutathione pull-downs [20], 150 ml culture pellets of yeast cells were split into two tubes and lysed using bead beating for 30 min. The lysis buffer contained 10 mM Tris (pH 7.5), 100 mM NaCl, 0.5 mM EDTA and 0.1% NP40. Post-bead beating, cells were spun at 5500rpm for 10 min at 4°C. The dilution buffer (10 mM Tris pH 7.5, 100 mM NaCl) was added to the supernatant. The input was collected from the mix and 50 µl of equilibrated glutathione sepharose 4B (GE healthcare) was added to the reaction mix. The reaction mixture was incubated for 2 hat 4°C followed by spin at 1500rpm for 30 s. The beads were washed two times using the dilution buffer. The beads were resuspended with 200 µl of dilution buffer. About 2.5% of Input and 5% PD were loaded on 8% SDS-PAGE gel. Ponceau was used as a loading control in all the blots unless specified. The remaining pull-down fraction was used for RNA isolation using TRIzol method. To determine the methylation status of Scd6, pGP564 EV and pGP564 Scd6GFP were transformed into BY4741. About 7.5 µl of equilibrated magnetic GFP trap beads (ChromoTek) was added to the reaction mix. The rest of the procedure for GFP trap pull-down was the same as described for the glutathione pull-down. Mono methyl arginine (MMA) antibody (Cell Signaling Technology) CST, catalogue (no. 8711; 1:1000 dilution) was used to detect the methylation signal of Scd6 with α-rabbit as the secondary antibody.

For Protein A pull-down, the cutures were collected as described for glutathione pull-down. The cultures were lysed using the lysis buffer mentioned for the glutathione pull-down. The lysate was incubated with anti‐Myc antibody for 1 h in binding buffer (50 mM Tris pH8, 150 mm NaCl and 0.15% NP40). Twenty microlitre of Protein A Beads (GE Healthcare) were added to each tube followed by incubation for 2 h of binding on the nutator at 4°C. The pellet was washed thrice with binding buffer, 5% of the pull-down fraction was resuspended in 1× laemmli buffer followed by boiling for 5 min at 100°C. Fifty percentage of the pellet was loaded on SDS/PAGE gel for western analysis to confirm the pull-down. The remainder of the pull-down fraction was used for RNA isolation followed by qRT-PCR for RNA Immunoprecipitation.

All the antibodies used in the study have been listed in Table 4.

**Table 4.** List of antibodies used in the study.

Antibody	Description
α-GFP	BioLegend, Catalog no. 338,001
α-mono methyl arginine	CST, catalogue no. 8711
α-PGK1	abcam, Catalogue no. ab113687
α-Mouse	Jackson ImmunoResearch Laboratories, Code No. 115- 035–003
α-Rabbit	Jackson ImmunoResearch Laboratories, Code No. 111- 035–003
α-GST	CST 26H1 Mouse mAb Catalogue no. 2624
α-Myc	Sigma, catalogue no. C3956
α- G3BP1	Santa Cruz Biotechnology,sc-365338
α-DCP1	Santa Cruz Biotechnology, sc-100706
Alexa Fluor^TM^ 568 goat α -Mouse	Invitrogen, catalogue no. A11004

### RNA isolation

Hot acidic phenol method: RNA isolation was performed using the hot acidic phenol method [46]. Mid-log phase cells were harvested by centrifugation. The cell pellet was resuspended in DEPC-treated autoclaved MQ. The cells were collected by a flash spin at 10k rpm. The cell pellet was resuspended in 400 μl of TES solution (10 mM Tris Chloride pH 7.5, 10 mM EDTA and 0.5% SDS). 400 μl of hot acidic phenol was added to the tube, followed by vigorous vortexing for 10 s. The tubes were incubated at 65°C for 60 min and vortexed every 15 min. After incubation, it was kept on ice for 5 min followed by spinning at 14,000rpm for 10 min at 4°C. The aqueous layer obtained by this step was transferred into another tube already having 400 μl of Chloroform. The mixture was then vortexed vigorously for 10 s. The tubes were spun at 14,000 rpm for 10 min, 4°C. The aqueous layer was then carefully transferred into another tube. To this tube, 1/10th vol of 3 M NaAc pH 5.2 and 2.5 vol of EtOH were added. This mixture was snap-chilled using liquid N2. The tubes were spun at 14000 rpm for 10 min at 4°C. The pellet was washed twice with 70% EtOH, air-dried, and resuspended using 100 μl DEPC MQ. RNA quality was checked by 1% agarose formamide gel electrophoresis. The RNA was used to assess *Scd6* and *CTT1* mRNA levels shown in Figures S3 C,D and E was isolated using the TES method protocol.

TRIzol method for RNA isolation: RNA was isolated from yeast samples using the TRIzol (G Biosciences, Cat. No. 786–652) and the chloroform extraction method. Briefly, lysates, pull-down, or polysomal fractions were mixed with 1 mL of TRIzol reagent and 200 µL of chloroform. After vigorous vortexing, samples were centrifuged at 14,000 rpm for 20 min. The aqueous phase was then subjected to isopropanol precipitation by flash freezing, followed by centrifugation at 15,000 rpm for 30 min at 4°C. The resulting RNA pellet was washed once with 70% ethanol at room temperature, air-dried, and resuspended in an appropriate volume of DEPC-treated Milli-Q water. RNA samples were then treated with DNase I (Thermo Scientific, Cat. No. EN0521) prior to cDNA synthesis. cDNA libraries were prepared from 1 µg of RNA using random primers. The resulting cDNA was diluted 1:10 for lysate samples and 1:1 for pull-down and polysome fractions and used as templates for RT-qPCR. Reactions were performed in 10 µL volumes for 35 cycles using TB Green Premix Ex Taq II (Tli RNase H Plus; TaKaRa, Cat. No. RR820B) using gene-specific primers (Bioserve).

### Reverse transcription and quantitative real-time PCR

The isolated RNA was treated with DNase I (Thermo, EN0525). 5 μg of total RNA, 2.5 units of DNase I, and DNase I Buffer with MgCl_2_ were added for a 30 μl reaction. The mixture was then incubated at 37°C for 30 min. After incubation, 3 μl 50 mM EDTA was added to stop the DNase I reaction. The DNase I-treated RNA was analysed using 1.2% agarose formamide gel electrophoresis to assess RNA quality. DNaseI treated RNA was then used for cDNA synthesis. According to the manufacturer’s protocol, 1 µg of RNA was used to synthesize cDNA using the RevertAid RT Reverse Transcription Kit (Thermo, K1691). cDNA was diluted at 1:10, and real-time PCR was performed using TB Green™ Premix Ex Taq™ (TaKaRa). For qRT-PCR, three technical replicates were assembled with 2 μl cDNA/reaction and 0.5 µM of each primer in a BioRad iQ5 Real-Time PCR Detection System. The PCR conditions were 95°C for 12 min for the initial denaturation, followed by 30 cycles of 95°C for 20 sec, 46°C for 30 sec, and 72°C for 30 s. The DNA was quantified in every cycle during extension. Melt curve acquisition was performed out at 64°C for 8 sec. Ct values were extracted using automatic baseline and manual thresholds. ΔΔCt method was used to calculate the final log2 Fold Change values, which were then plotted on a 19 box and whisker plot using GraphPad Prism 8.0.

For quantification of relative mRNA enrichment in RIP and polysome profiling assays, ∆Ct values for each target gene were calculated by subtracting the Ct value of the internal control gene PGK1 from the Ct value of the gene of interest. Subsequently, ∆∆Ct values were determined by subtracting the ∆Ct of total RNA from the ∆Ct of immunoprecipitated (pull-down) RNA. Finally, the relative enrichment values were obtained by normalizing 2^(−∆∆Ct)^ to the corresponding Scd6 pull-down signal intensities.

### smFISH

Wild-type (WT) yeast cells were diluted from an overnight primary culture to an initial optical density (OD_6__00_) of 0.1 and grown to an OD_6__00_ of 0.5. The cells were treated with H_2_O_2_ for 30 min in the dark conditions. Following H_2_O_2_ treatment, cells were fixed with 3.7% paraformaldehyde (PFA) for 45 min at room temperature. The fixed cells were washed with cold Buffer B (1.2 M sorbitol and 100 mM KHPO_4_, pH 7.5, prepared in nuclease-free water). For spheroplasting, the fixed cells were incubated in a spheroplasting buffer, consisting of 890 µL Buffer B, 100 µL of 200 mM VRC, 10 µL of 25 kU/mL lyticase, and 2 µL β-mercaptoethanol, for 5 min at 30°C. Post-spheroplasting, after which the cells were washed again with Buffer B and incubated in 70% ethanol for 4 h at −20°C.

Approximately 50 µL of cells was washed twice with Stellaris wash buffer (2X SSC, 10% formamide [deionized] in nuclease-free water). CTT1-specific probes (Table 5) were annealed to secondary FLAPX A647 (CCTCCTAAGTTTCGAGCTGGACTCAGTG) probe (Cy5-labelled). The annealing reaction contained 200 pmol of CTT1-specific probe, 250 pmol of FLAP probe, and 10X NEB Buffer 3 in nuclease-free water. Next, 3 µL of the FLAP-CTT1 probe mix was added to the smFISH hybridization buffer, composed of 10 µg/mL *E. coli* tRNA, 100 µL of 200 mM VRC, 200 µL of 10 mg/mL BSA, 1 mL of 20X SSC, 2 mL of 50% dextran sulphate, and 10% formamide. Hybridization mix was added to the cells, which were incubated overnight on a nutator at 37°C in the dark.

**Table 5.** List of smiFISH probes for CTT1.

Probe #	Probe (5’- > 3’) with 5 Flap X sequence at 3’ end	Probe position *
1	gctgattgatcttattggcaCCTCCTAAGTTTCGAGCTGGACTCAGTG	80
2	gcttttcttcttttttaccgCCTCCTAAGTTTCGAGCTGGACTCAGTG	123
3	atgggtgatgagagtacggaCCTCCTAAGTTTCGAGCTGGACTCAGTG	174
4	cgtctggtcttgagtattgaCCTCCTAAGTTTCGAGCTGGACTCAGTG	201
5	ttttccagcagatggaagtcCCTCCTAAGTTTCGAGCTGGACTCAGTG	238
6	cggaactctttctctatcgaCCTCCTAAGTTTCGAGCTGGACTCAGTG	269
7	gttcgaactccagtctacaaCCTCCTAAGTTTCGAGCTGGACTCAGTG	318
8	atggagcggcgtatgtaataCCTCCTAAGTTTCGAGCTGGACTCAGTG	357
9	ggacatttgtaacccacattCCTCCTAAGTTTCGAGCTGGACTCAGTG	382
10	tcaccaccaacggtggaaaaCCTCCTAAGTTTCGAGCTGGACTCAGTG	415
11	aagaaacacctcttgggtctCCTCCTAAGTTTCGAGCTGGACTCAGTG	459
12	attgttgaagacccagtcatCCTCCTAAGTTTCGAGCTGGACTCAGTG	506
13	gcgtctctgaggaagaagacCCTCCTAAGTTTCGAGCTGGACTCAGTG	532
14	ttcagatgagactgagggtcCCTCCTAAGTTTCGAGCTGGACTCAGTG	589
15	cagtatatggtagtgtcctgCCTCCTAAGTTTCGAGCTGGACTCAGTG	616
16	atggattgattccggattcaCCTCCTAAGTTTCGAGCTGGACTCAGTG	650
17	acctctatcaccaaacatgtCCTCCTAAGTTTCGAGCTGGACTCAGTG	680
18	cagagtacgcgttcatactaCCTCCTAAGTTTCGAGCTGGACTCAGTG	717
19	taccttctttgttgaccatgCCTCCTAAGTTTCGAGCTGGACTCAGTG	750
20	gtatccgacaagacgtggaaCCTCCTAAGTTTCGAGCTGGACTCAGTG	787
21	atctccagtcaaggtttcaaCCTCCTAAGTTTCGAGCTGGACTCAGTG	812
22	tgaacagctttgcctgattaCCTCCTAAGTTTCGAGCTGGACTCAGTG	867
23	cttttcgccattttgcaattCCTCCTAAGTTTCGAGCTGGACTCAGTG	890
24	ggtgtcattgtttgcacataCCTCCTAAGTTTCGAGCTGGACTCAGTG	925
25	cctgaacttagttgcttgttCCTCCTAAGTTTCGAGCTGGACTCAGTG	947
26	tgtggccatattttcgttagCCTCCTAAGTTTCGAGCTGGACTCAGTG	982
27	gttagggtgatggtaccaaaCCTCCTAAGTTTCGAGCTGGACTCAGTG	1024
28	tgttcgttggactgaatgcaCCTCCTAAGTTTCGAGCTGGACTCAGTG	1083
29	agaaggcttaatacctgggaCCTCCTAAGTTTCGAGCTGGACTCAGTG	1109
30	aagtctggcttgtagaacggCCTCCTAAGTTTCGAGCTGGACTCAGTG	1136
31	ctatgacgttgagtgtctggCCTCCTAAGTTTCGAGCTGGACTCAGTG	1165
32	ttgacgggcaattgctgataCCTCCTAAGTTTCGAGCTGGACTCAGTG	1198
33	agtatggacatcccaagtttCCTCCTAAGTTTCGAGCTGGACTCAGTG	1227
34	gtgtattgggaatcacctttCCTCCTAAGTTTCGAGCTGGACTCAGTG	1249
35	ctttggaagttcactgctttCCTCCTAAGTTTCGAGCTGGACTCAGTG	1288
36	taatttggctcaggaccgaaCCTCCTAAGTTTCGAGCTGGACTCAGTG	1333
37	agatacttcgtcgttgtcttCCTCCTAAGTTTCGAGCTGGACTCAGTG	1394
38	tcgtcaagaactatccctttCCTCCTAAGTTTCGAGCTGGACTCAGTG	1423
39	gttcctgttttctcacagaaCCTCCTAAGTTTCGAGCTGGACTCAGTG	1455
40	ggcatcaacaatatgctcgtCCTCCTAAGTTTCGAGCTGGACTCAGTG	1490
41	tcatatagagctcttggctgCCTCCTAAGTTTCGAGCTGGACTCAGTG	1561
42	tctgttcatcgttgtataccCCTCCTAAGTTTCGAGCTGGACTCAGTG	1584
43	tttgatcttacaagcgtggcCCTCCTAAGTTTCGAGCTGGACTCAGTG	1631
44	gcgtaactctctttttgactCCTCCTAAGTTTCGAGCTGGACTCAGTG	1659
45	cccaaatcttcgtttagcaaCCTCCTAAGTTTCGAGCTGGACTCAGTG	1690
46	ccaagcattctgcaatgactCCTCCTAAGTTTCGAGCTGGACTCAGTG	1713
47	aaccttcaaggtcaacaggtCCTCCTAAGTTTCGAGCTGGACTCAGTG	1746
48	ccttaattggcacttgcaatCCTCCTAAGTTTCGAGCTGGACTCAGTG	1783

Following hybridization, the cells were centrifuged and washed with Stellaris wash buffer for 30 min at 37°C in a nutator. This was followed by a wash with 2X SSC +0.1% Triton-X 100 at room temperature (RT) for 15 min and a final wash with 1X SSC for 15 min with gentle rocking at RT. The cells were resuspended in 1X PBS and mounted on coverslips. DAPI stain was added to the Fluoromount-G mounting reagent. The slides were allowed to rest for 4–5 h before imaging. Images were obtained using a DeltaVision RT microscope. Thirty cells counted for each condition for quantification shown in Figure 5D. To assess the relative association of Scd6 with the *CTT1* mRNA under unstressed conditions, we measured the raw integrated density of *CTT1* mRNA puncta and the corresponding Scd6 signal at the same spatial coordinates. The Scd6 intensity was then normalized to the CTT1 mRNA intensity, and the resulting ratio was plotted on the Y-axis to reflect the relative enrichment of Scd6 at *CTT*1 mRNA puncta (Figure 4 E iv).

### Cell culture and transfections

Glass coverslips were pre-coated with poly-L-lysine (0.05 mg/ml) for 2 h followed by three PBS washes. HeLa cells (procured from cell repository, NCCS Pune) were cultured in Dulbecco’s Modified Eagle Medium (DMEM, Gibco) with 1X antibiotic-antimycotic (Thermo, 15,240,062), 10% FBS (foetal bovine serum, Gibco, A52567), and 5% CO2 at 37°C on pre-coated glass coverslips. Mycoplasma contamination was checked once every month using the PCR-based approach as described earlier [47] or transfections of eGFP and LSm14AGFP plasmids, Lipofectamine 2000 reagent (Invitrogen) was used (as per manufacturer’s protocol) followed by treatment of cells with H_2_O_2_ at 1 mM concentration for 2 h.

## MTT assay

HeLa cells were seeded in a 12-well plate in DMEM medium with 1X antibiotic-antimycotic (Thermo, 15,240,062), 10% FBS, and 5% CO_2_ at 37°C. Twenty-four h post transfections with eGFP and LSm14AGFP plasmids, cells were subjected to 2 mM H_2_O_2_ treatment for 2 h. HeLa cells were washed thrice with 1X PBS and MTT 3-[4,5-dimethylthiazol-2-yl]-2,5-diphenyltetrazolium bromide (MTT) reagent (5 mg/ml) was then added to cells and kept at 37°C for 2 h in CO_2_ incubator. After 2 h of incubation, MTT containing media was discarded, and 300 μl of DMSO was added to each well. The plate was then kept at 37°C for 15 min to solubilize the formazan crystals, and the absorbance was measured using a microplate reader at a wavelength of 570 nm.

## Immunocytochemistry

HeLa cells were treated with H_2_O_2_ treatment (1 mM for 2 h and autoclaved MQ as vehicle control), fixed with formaldehyde and permeabilized with permeabilization buffer (1X PBS with 0.13% Triton® X-100 0694-1 L, VWR) for 20 min at room temperature. Cells were then washed thrice with 1X PBS followed by blocking with 1% BSA and 0.3% Triton® X-100 in 1X PBS for 2 h at room temperature. Cover slips were incubated overnight at 4°C with primary antibodies (anti G3BP1, sc-365338, Santa Cruz Biotechnology and anti-DCP1a, sc-100706, Santa Cruz Biotechnology, dilution 1:100 in blocking solution) in humid chamber followed by three washes with PBST. The cells were then incubated with Alexa Fluor^TM^ 568 goat anti-mouse secondary antibody (Invitrogen), diluted 1:300 in blocking solution followed by three washes with PBST. Nucleus was stained with DAPI (D1306, Thermo) for 20 min at room temperature, and coverslips were mounted on glass slides using Fluoromount-G (00–4958-02, Thermo). G3BP1 and DCP1a signals were captured using the Deltavision RT microscope system running softWoRx 3,5,1 software and imaging was performed as described earlier. The exposure time and transmittance settings for FITC (Fluorescein isothiocyanate) and TRITC to detect GFP-tagged protein and G3BP1 and Dcp1a were 0.2 s and 10% and 0.5% and 10%, respectively, for HeLa cells. For DAPI, the exposure time and transmittance settings were 0.1 s and 2%, respectively. All images were deconvoluted using standard softWoRx deconvolution algorithms. Images were analysed in ImageJ software. For each experiment, GFP-tagged LSm14A puncta from 30 cells were counted for quantification. Statistical significance was calculated using the paired t-test.

## Supplementary Material

Supplementaryfigures1to3_Tiwarietal2025.docx

Supplementaryfigures4to6_Tiwarietal2025.docx

## Data Availability

The authors confirm that the data supporting the findings of this study are available in the article and its supplementary materials. Any further underlying data will be made available upon reasonable request.
