# Supplementary material for: RGG-motif protein Scd6 affects oxidative stress response by regulating cytosolic caTalase T1 (Ctt1)
Source: RNA Biol. 2026 Jan 9;23(1):1–23. doi: 10.1080/15476286.2026.2613892 (PMC12795261; doi:10.1080/15476286.2026.2613892)
Supplement: Supplementaryfigures1to3_Tiwarietal2025.docx [file KRNB_A_2613892_SM0118.docx]

**RGG-motif protein Scd6 affects oxidative stress response by regulating Cytosolic caTalase T1 (Ctt1)**

**Tiwari et al, 2025**

**Supplementary figures-1-3**

**Untreated**

**H_2_O_2_**

**H_2_O_2_ +CHX**

**Scd6GFP puncta/cell**

**Recovery**

**(A)**


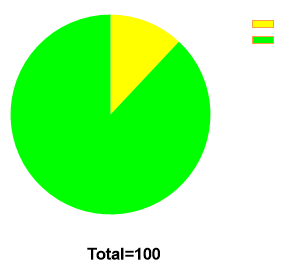


**12%**

**88%**

**Edc3+Scd6**

**Scd6**

**H_2_O_2_**

**(B)**

**Scd6**

**DIC**

**Untreated**

**H_2_O_2_**


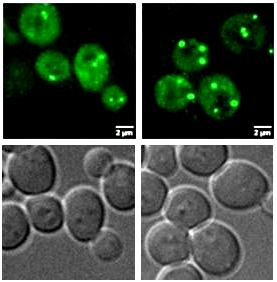


**30 minutes**

**60 minutes**

**90 minutes**

**0 minutes**

**Untreated**

**H_2_O_2_**

**DIC**

**Scd6**

**Pab1**


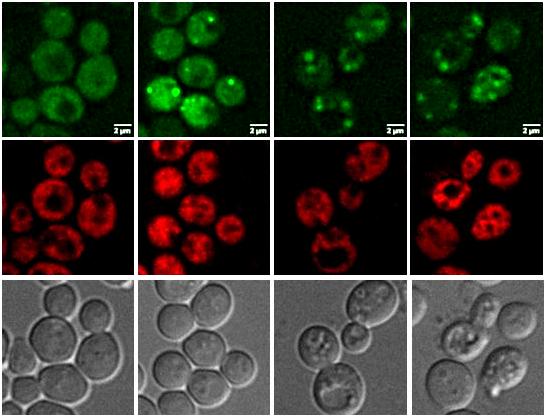


**Untreated**

**H_2_O_2_**

**Edc3mCh puncta/cell**

**Scd6GFP puncta/cell**

**Untreated**

**H_2_O_2_**

**Scd6GFP puncta/cell**

**Untreated**

**H_2_O_2_**

**(C)**

**(D)**

**(E)**

**(F)**

**(G)**

**(H)**

**DIC**

**Scd6GFP**

**LSm1mCh**

**Merge**

**Untreated**

**H_2_O_2_**


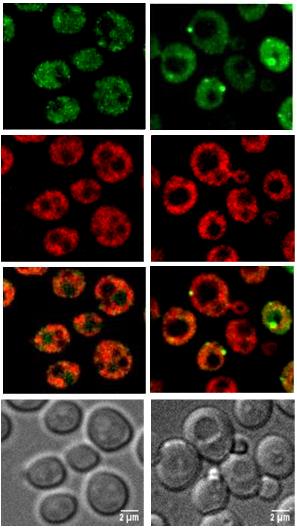


**DIC**

**Scd6GFP**

**Pub1mCh**

**Merge**

**Untreated**

**H_2_O_2_**

**Figure S1: Scd6 localizes to cytoplasmic puncta upon H_2_O_2_ stress-** (A) Quantification for the Scd6 puncta formation in plasmid in untreated, 4mM H_2_O_2,_CHX and recovery conditions (60 minutes) where data plots represent **mean ± SD** from *n* = 3, where ‘*n*’ represents number of independent experiments. (B) Live cell imaging of WT cells expressing Scd6GFP under untreated and 4mM H_2_O_2,_ treated condition (C) Quantification for the Scd6 granule formation in (B) (Projected image has been presented here). (D) Quantification for Scd6GFP, Edc3mCherry puncta for Figure 1C representing **mean ± SD** from *n* = 3. (E) pie chart depicting the % colocalization of Edc3 with Scd6 upon H_2_O_2_  stress. Two-tailed paired t-test was used to calculate the statistical significance (F) Live cell imaging of Pab1-RFP expressing Scd6GFP plasmid under untreated and 4mM H_2_O_2_ treated condition for 30, 60 and 90 minutes of treatment. (G) Live cell imaging for Pub1mCh transformed into Scd6GFP strain plasmid under untreated and 4mM H_2_O_2_ treated condition (30 minutes). (H) Live cell imaging for LSm1mCh transformed into Scd6GFP strain plasmid under untreated and 4mM H_2_O_2_ treated condition (30 minutes). Asterisks indicate levels of statistical significance: *p* < 0.05 (*), *p* < 0.01 (**), *p* < 0.001 (***), and *p* < 0.0001 (****).

**Figure S2: Alteration of Scd6 levels affects survival upon H_2_O_2_ stress**-Growth curve analysis for mid-log phase WT/*scd6∆/ctt1 ∆* cells treated with varying concentrations of H_2_O_2_ (A) 1mM (B) 2mM (C) 3mM for 30 minutes. (p<0.0001 for WT vs. WT H_2_O_2,_ *scd6∆* VS vs. *scd6∆* H_2_O_2_ and *ctt1∆* vs. *ctt1∆* H_2_O_2_). Growth curve analysis for mid-log phase WT cells transformed with EV/ Scd6GST/ Scd6GST∆RGG/ctt1 ∆EV treated with varying concentrations of H_2_O_2_ (D) 1mM (E) 2mM (F) 3mM for 30 minutes (p<0.0001 for EV vs. EV H_2_O_2,_  Scd6GST vs. Scd6GST H_2_O_2,_ Scd6 *∆*RGG vs. Scd6 *∆*RGG H_2_O_2_ and *ctt1∆*vs. *ctt1∆*H_2_O_2_ ). Growth curves shown represent mean ± SEM from *n* =4 , where ‘*n*’ represents number of independent experiments. Tukey’s test for variance was used to calculate the statistical significance ‘*’. Asterisks indicate levels of statistical significance: *p* < 0.05 (*), *p* < 0.01 (**), *p* < 0.001 (***), and *p* < 0.0001 (****).

**(A)**

**(B)**


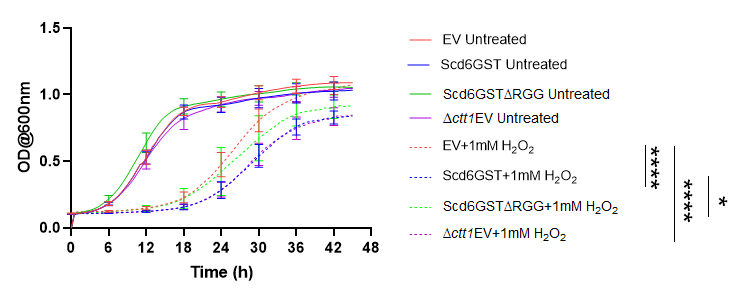

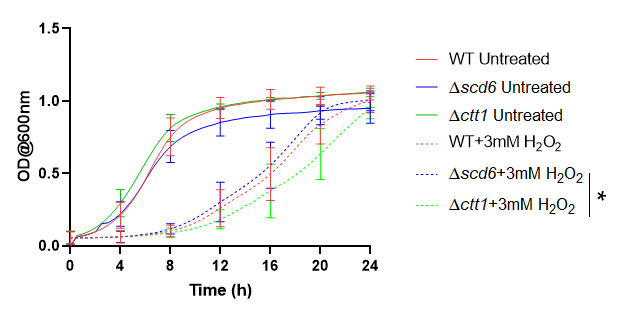

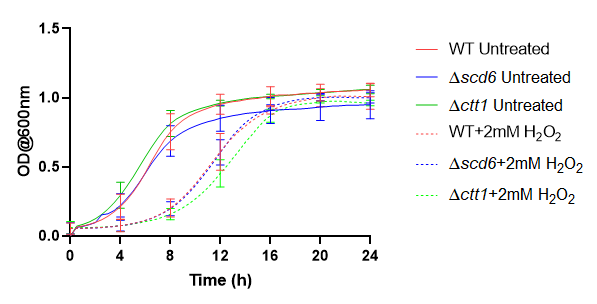

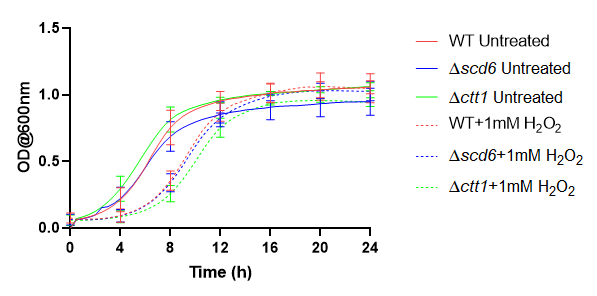


**(C)**

**(D)**

**WT**

***scd6∆***

***ctt1∆***

**WT**

***scd6∆***

***ctt1∆***

**Untreated**

**1mM H_2_O_2_**

**WT**

***scd6∆***

***ctt1∆***

**WT**

***scd6∆***

***ctt1∆***

**Untreated**

**2mM H_2_O_2_**

**WT**

***scd6∆***

***ctt1∆***

**WT**

***scd6∆***

***ctt1∆***

**Untreated**

**3mM H_2_O_2_**

**EV**

**Scd6**

***ctt1∆***

**EV**

**Scd6**

***ctt1∆***

**RGG*∆***

**RGG*∆***

**Untreated**

**1mM H_2_O_2_**

**Time (h)**

**OD@600nm**

**Time (h)**

**OD@600nm**

**Time (h)**

**OD@600nm**

**Time (h)**

**OD@600nm**

**Time (h)**


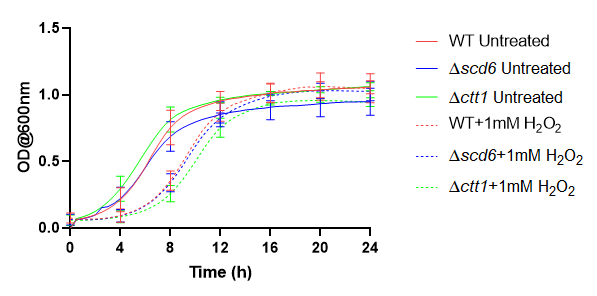

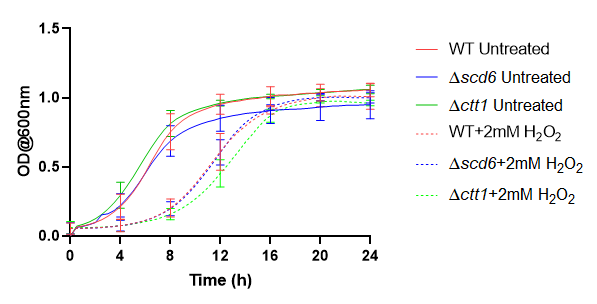

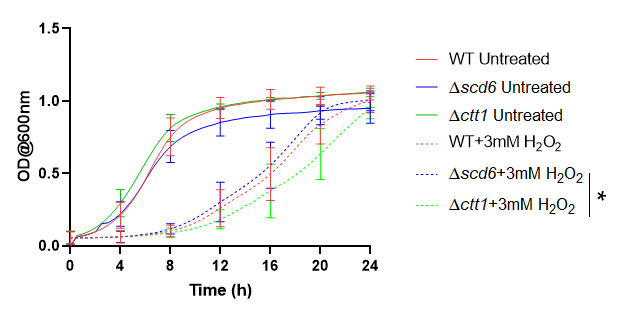

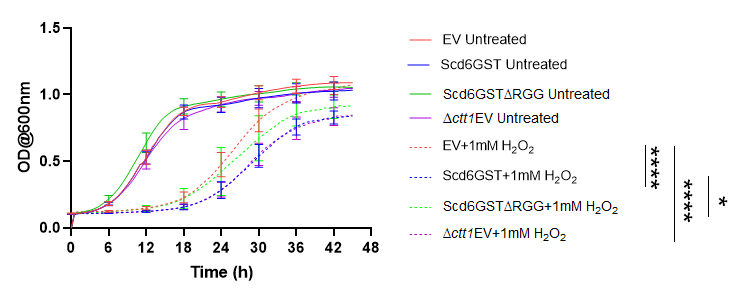

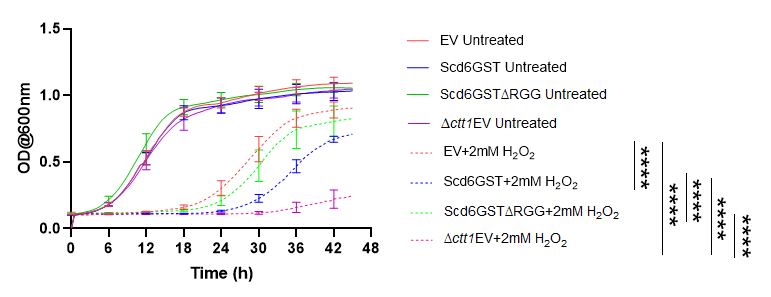


**(E)**

**EV**

**Scd6**

***ctt1∆***

**EV**

**Scd6**

***ctt1∆***

**RGG*∆***

**RGG*∆***

**Untreated**

**2mM H_2_O_2_**

**OD@600nm**


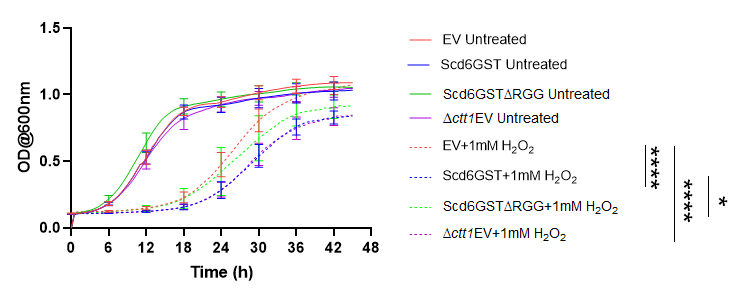


****

****


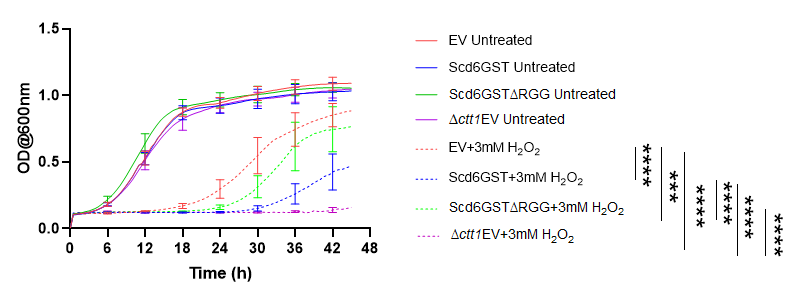


**(F)**

**EV**

**Scd6**

***ctt1∆***

**EV**

**Scd6**

***ctt1∆***

**RGG*∆***

**RGG*∆***

**Untreated**

**3mM H_2_O_2_**

**OD@600nm**

**Time (h)**


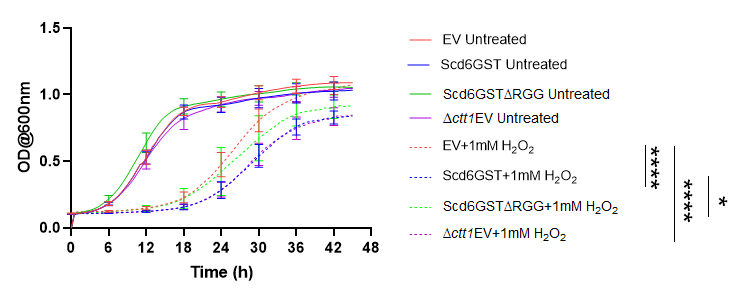


****

****

***

****

*

**Figure S3**: (A)Absolute quantification for Ctt1-GFP protein levels in *scd6∆* for Figure 3B and (B) Log2FC for *CTT1* mRNA levels in *scd6∆* under untreated and 4mM H_2_O_2_ treated condition for 30 minutes. (C) Log2FC relative Scd6 mRNA levels for EV and Scd6GST (D) The blot represents Scd6myc protein levels in Scd6myc strains transformed with EV and Scd6GST. The overexpression of Scd6GST was confirmed using α-GST antibody. The Scd6myc signal was detected by α-myc antibody. Ponceau was used as a loading control. (*E*) Absolute quantification for Ctt1-GFP protein levels in Scd6 overexpression for Figure 3D and (F) Log2FC relative Scd6 mRNA levels for EV and Scd6GST in untreated and 4mM H_2_O_2_ treated condition. (G) Absolute protein levels for Scd6GST/Scd6GST∆RGG overexpression upon 4mM H_2_O_2_ treatment from Figure 3E. Data plots represent mean ± SEM where ‘*n*’ represents number of independent experiments. Two tailed paired student’s t- test was used to calculate the statistical significance ‘*’. Asterisks indicate levels of statistical significance: *p* < 0.05 (*), *p* < 0.01 (**), *p* < 0.001 (***), and *p* < 0.0001 (****).

**(A)**

**Scd6myc**

**70**


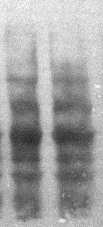

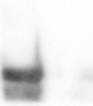

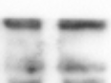


**Scd6GST**

**EV**

**Scd6GST**

**70**

**Ponceau**

**50**

**(B)**


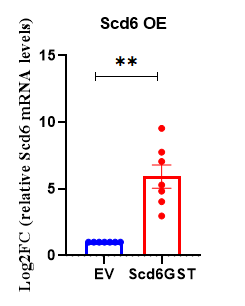


**EV**

**Scd6**

**Log2FC (Scd6 mRNA levels )**


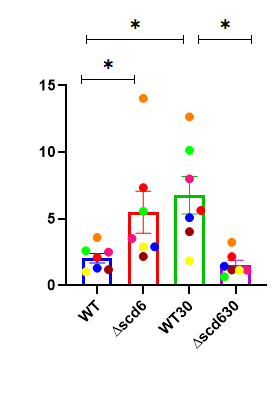


**(C)**

**Ctt1 protein levels**

**WT**

***scd6∆***

**WT**

***scd6∆***

**Untreated H_2_O_2_**

**WT**

***scd6*∆**


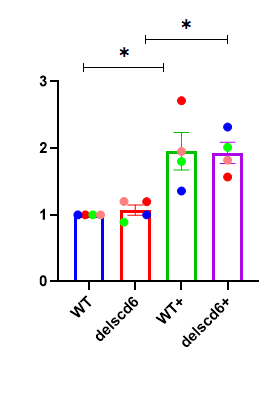


**WT**

***scd6∆***

**Untreated H_2_O_2_**

**Log2FC (*CTT1* mRNA levels)**

**(D)**


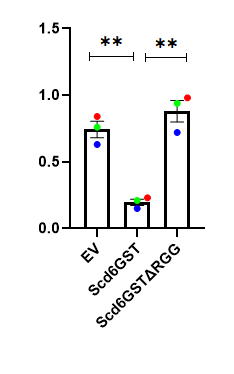


**Ctt1 protein levels**

**EV**

**Scd6∆RGG**

**Scd6**

**(E)**

**Untreated H_2_O_2_**

**Log2FC (*CTT1* mRNA levels)**

**EV**

**Scd6**

**EV**

**Scd6**


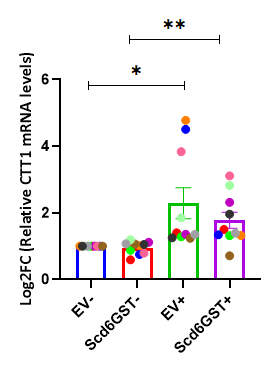


**Ctt1 protein levels**


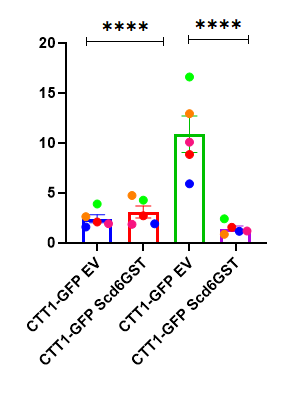


**EV**

**Scd6**

**EV**

**Scd6**

**Untreated H_2_O_2_**

**(F)**

**(G)**
