# Supplementary material for: RGG-motif protein Scd6 affects oxidative stress response by regulating cytosolic caTalase T1 (Ctt1)
Source: RNA Biol. 2026 Jan 9;23(1):1–23. doi: 10.1080/15476286.2026.2613892 (PMC12795261; doi:10.1080/15476286.2026.2613892)

**RGG-motif protein Scd6 affects oxidative stress response by regulating Cytosolic caTalase T1 (Ctt1)**

**Tiwari et al, 2025**

**Supplementary figures-4 and 5**

**Figure S4**: **Scd6 Interacts with *CTT1* in unstressed conditions and dissociates upon H_2_O_2_ stress-induced granule formation -**(A) Log₂ fold-change (Log2FC) for *CTT1* in the PD fraction showing the absolute values for the enrichment of *CTT1* mRNA in the Scd6myc pull-down (Untreated and H_2_O_2_ treated) shown in Figure 5. One-way ANOVA was used to calculate the statistical significance ‘*’. Log₂ fold-change (Log2FC) for (B) *RAD50* (C) *ACT1* in the Scd6myc pull-down (Untreated). Data plots represent mean ± SEM ( *n* = 3, where ‘*n*’ represents number of independent experiments).Two tailed paired student’s t- test was used to calculate the statistical significance ‘*’(D) Blot representing Scd6myc protein levels in untreated and H_2_O_2_ treated condition. Scd6 signal is detected using α-myc antibody. Ponceau was used as a control for normalization (E) Quantification for (D). (F) *ctt1∆* was used a control for smiFISH experiments to ensure the specificity of CTT1 specific smiFISH-probes. Asterisks indicate levels of statistical significance: *p* < 0.05 (*), *p* < 0.01 (**) and *p* < 0.001 (***).


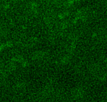

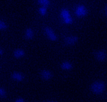

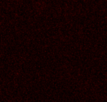


***CTT*1**

**GFP**

**DAPI**

**∆*ctt1***

**(D)**


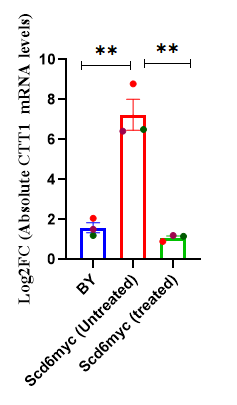

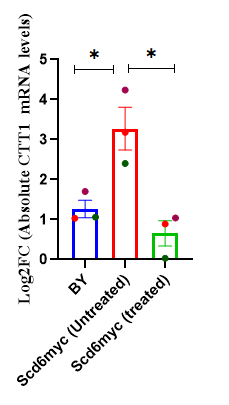


**Scd6+ H_2_O_2_**

**Untagged**

**Scd6**

**Log2FC (Absolute *CTT1* mRNA levels)**

**Scd6+ H_2_O_2_**

**Untagged**

**Scd6**

**Log2FC ( Absolute *CTT1* mRNA levels)**

**Primer 1**

**Primer 2**

**Untagged**

**Scd6**

**Log2FC Absolute mRNA levels)**

***RAD50***

**Untagged**

**Scd6**

**Log2FC (Absolute mRNA levels)**

***ACT1***

**(A)**

**(B)**

**(C)**


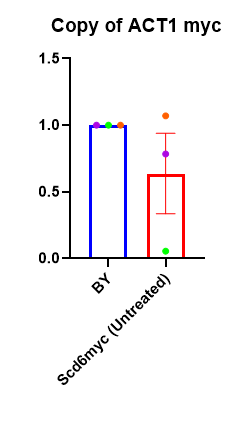

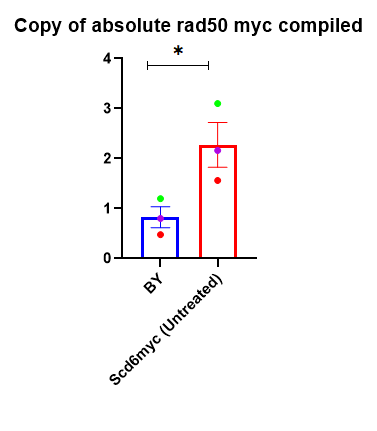

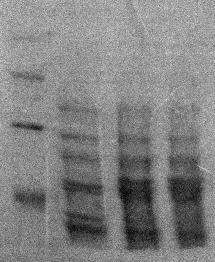

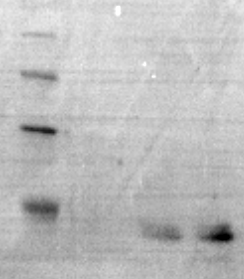


**Scd6**

**Scd6+H_2_O_2_**

**Scd6myc**

**Ponceau**


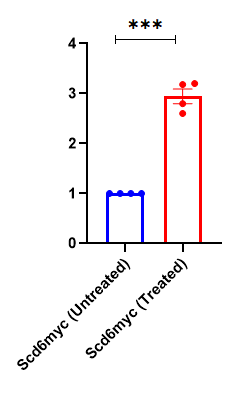


**Scd6**

**Scd6+H_2_O_2_**

**Scd6 protein levels**

**(D)**

**(E)**

**(F)**

**Figure S5**: **Overexpression from 2µ plasmid drives the interaction of Scd6 with *CTT1* mRNA -** (A) Schematic for primer positions on CTT1 (B) Blot represents Input and PD for Scd6GST pull-down in untreated and H_2_O_2_ treated condition. The GST signal was detected using α-GST antibody. PGK1 was used as a control. (C) Log₂ fold-change (Log2FC) for *CTT1* in the PD fraction showing the enrichment of *CTT1* mRNA in the Scd6GST pull-down for Primer 1 and Primer 2. (D) Polysome profile analysis of WT cells transformed with Scd6GST under untreated and H₂O₂-treated conditions. The values shown are normalized to the untreated WT cells (Dotted line represents the WT). Log₂ fold-change (Log2FC) for (E) *CTA1* (F) *RAD50* (G) *ACT1* in Scd6GST PD fraction (Untreated). Data plots represent mean ± SEM ( *n* = 7) where ‘*n*’ represents number of independent experiments. (H) Blot represents Input and pull down for Scd6GST/Scd6GST∆RGG (untreated). The GST signal was detected using α-GST antibody. PGK1 was used as a control. (I) Log₂ fold-change (Log2FC) for *CTT1* in the PD fraction showing the enrichment of *CTT1* mRNA in the Scd6GST /Scd6GST∆RGG pull-down for Primer 1 and Primer 2. Statistical significance was assessed using a one-way ANOVA. Asterisks indicate levels of statistical significance: *p* < 0.05 (*), *p* < 0.01 (**), *p* < 0.001 (***), and *p* < 0.0001 (****).

**(A)**

**(B)**

**(D)**

**Scd6**

**Primer 2F**

**Primer 2R**

**Primer 1F**

**Primer 1R**

**5’UTR**

**3’UTR**

**ORF**

**(G)**

**Log2FC CTT1**

**EV**

**Scd6∆RGG**

**Scd6**

**Primer 1**

**EV**

**Log2FC *CTT1***

**Scd6∆RGG**

**Primer 2**

**(C)**

**Scd6+ H_2_O_2_**

**EV**

**Scd6**

**Log2FC *CTT1***

**Primer 2**

**Scd6**

**Scd6∆RGG**

**Scd6**

**Scd6∆RGG**

**Input (1%) PD (5%)**

**Pgk1**

**EV**

**EV**

**Scd6**


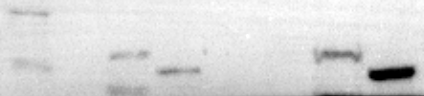

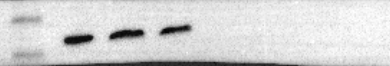


**70**

**50**

**Scd6 ∆RGG**


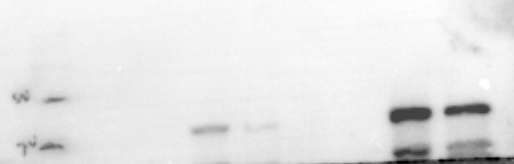


**Input (1%) PD (5%)**

**Scd6**

**EV**

**Scd6**

**Scd6+ H_2_O_2_**

**EV**

**Scd6**

**Scd6 + H_2_O_2_**

**Pgk1**

**70**

**50**


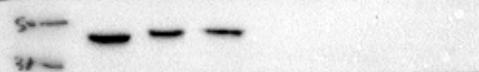


**Scd6+ H_2_O_2_**

**EV**

**Scd6**

**Log2FC *CTT1***


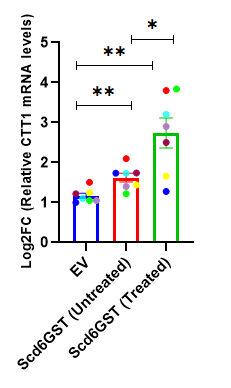

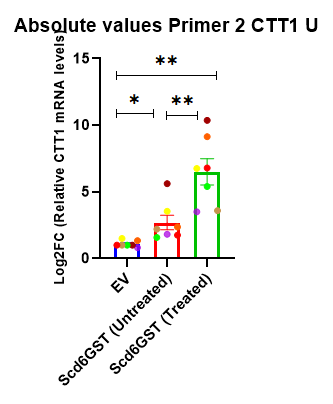

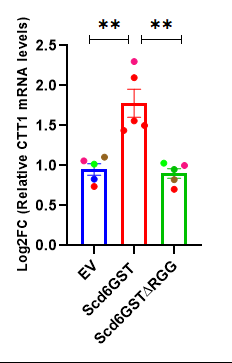

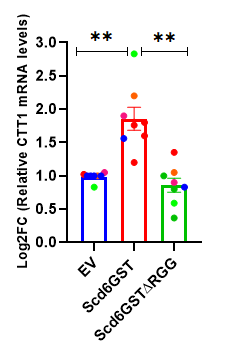


***RAD50***

**Log2FC**

**EV**

**Scd6**

***ACT1***

**Log2FC**

**EV**

**Scd6**

***CTA1***

**Log2FC**

**EV**

**Scd6**

**Primer 1**

**(E)**

**(F)**

**(H)**

**(I)**

**Log2FC *CTT1* mRNA levels**

**(Translated/untranslated)**

**Scd6+ H_2_O_2_**

**Scd6**

**Figure** **S6 : The methylation of Scd6 decreases upon H_2_O_2_ treatment-**Scd6GFP was pulled down using a GFP-Trap followed by probing with mono methyl arginine (MMA) and α-GFP antibody (A) MMA levels of Scd6GFP in untreated and H_2_O_2_ treated condition (B) Quantification for the MMA signal normalized with GFP levels. Data plots represent mean ± SEM from  *n* = 3, where ‘*n*’ represents number of independent experiments. Asterisks indicate level of statistical significance :*p* < 0.01 (**).

**Scd6GFP**

**α GFP**

**α MMA**

**EV**

**Untreated**

**H_2_O_2_**

**PD**

**(A)**

**(B)**


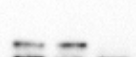


**75**


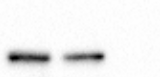


**75**

**MMA signal normalised**

**w.r.t to GFP signal**

**Untreated**

**H_2_O_2_**


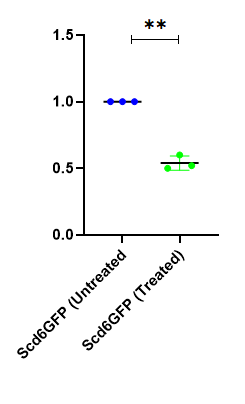

Supplement: Supplementaryfigures4to6_Tiwarietal2025.docx [file KRNB_A_2613892_SM0117.docx]
